# Evidence for Shared Genetic Aetiology Between Schizophrenia, Cardiometabolic, and Inflammation-Related Traits: Genetic Correlation and Colocalization Analyses

**DOI:** 10.1093/schizbullopen/sgac001

**Published:** 2022-01-11

**Authors:** Benjamin I Perry, Nicholas Bowker, Stephen Burgess, Nicholas J Wareham, Rachel Upthegrove, Peter B Jones, Claudia Langenberg, Golam M Khandaker

**Affiliations:** 1 Department of Psychiatry, University of Cambridge School of Clinical Medicine, Cambridge, UK; 2 Cambridgeshire and Peterborough NHS Foundation Trust, Cambridge, UK; 3 MRC Epidemiology Unit, University of Cambridge School of Clinical Medicine, Cambridge, UK; 4 MRC Biostatistics Unit, University of Cambridge, Cambridge, UK; 5 Institute for Mental Health, University of Birmingham, Birmingham, UK

**Keywords:** schizophrenia, cardiometabolic disorders, genetic, common aetiology, correlation, colocalization

## Abstract

**Background:**

Schizophrenia commonly co-occurs with cardiometabolic and inflammation-related traits. It is unclear to what extent the comorbidity could be explained by shared genetic aetiology.

**Methods:**

We used GWAS data to estimate shared genetic aetiology between schizophrenia, cardiometabolic, and inflammation-related traits: fasting insulin (FI), fasting glucose, glycated haemoglobin, glucose tolerance, type 2 diabetes (T2D), lipids, body mass index (BMI), coronary artery disease (CAD), and C-reactive protein (CRP). We examined genome-wide correlation using linkage disequilibrium score regression (LDSC); stratified by minor-allele frequency using genetic covariance analyzer (GNOVA); then refined to locus-level using heritability estimation from summary statistics (ρ-HESS). Regions with local correlation were used in hypothesis prioritization multi-trait colocalization to examine for colocalisation, implying common genetic aetiology.

**Results:**

We found evidence for weak genome-wide negative correlation of schizophrenia with T2D (r_g_ = −0.07; 95% C.I., −0.03,0.12; *P* = .002) and BMI (r_g_ = −0.09; 95% C.I., −0.06, −0.12; *P* = 1.83 × 10^−5^). We found a trend of evidence for positive genetic correlation between schizophrenia and cardiometabolic traits confined to lower-frequency variants. This was underpinned by 85 regions of locus-level correlation with evidence of opposing mechanisms. Ten loci showed strong evidence of colocalization. Four of those (rs6265 (*BDNF*); rs8192675 (*SLC2A2*); rs3800229 (*FOXO3*); rs17514846 (*FURIN*)) are implicated in brain-derived neurotrophic factor (BDNF)-related pathways.

**Conclusions:**

LDSC may lead to downwardly-biased genetic correlation estimates between schizophrenia, cardiometabolic, and inflammation-related traits. Common genetic aetiology for these traits could be confined to lower-frequency common variants and involve opposing mechanisms. Genes related to BDNF and glucose transport amongst others may partly explain the comorbidity between schizophrenia and cardiometabolic disorders.

## Introduction

People with schizophrenia die on average 10–15 years sooner than the general population,^1^ and physical comorbidity, including up to a 50% higher prevalence of cardiometabolic disorders, is a leading cause for the excess mortality.^2^ A better understanding of the mechanisms underlying the comorbidity is pivotal to inform novel approaches to treatment and prevention, and may help to close the mortality gap faced by people who have schizophrenia. Traditionally, the increased cardiometabolic risk observed in schizophrenia has been attributed solely to sociodemographic and lifestyle factors, and the adverse effects of antipsychotic medication.^3^ However, cardiometabolic dysfunction is detectable in antipsychotic-naïve young-adults with first-episode psychosis, suggesting that lifestyle factors/medication may not be the full explanation.^4^ For example, schizophrenia and cardiometabolic disorders share similar associations with elevated concentrations of circulating inflammatory markers such as C-reactive protein (CRP) and interleukin-6 (IL-6), both cross-sectionally^5,6^ and longitudinally.^7,8^ Mendelian randomization (MR) studies have shown that genetically-predicted levels of IL-6 and CRP could be causally linked with cardiometabolic disorders^9^ and schizophrenia,^10^ and that inflammation could be a common cause for comorbid insulin resistance and schizophrenia.^11^ Therefore, it is possible that schizophrenia, cardiometabolic, and inflammation-related traits could share pathophysiologic mechanisms, including a common genetic basis.

Previous studies have predominantly used Linkage Disequilibrium (LD) score regression analysis (LDSC)^12^ to estimate whole-genome correlation between schizophrenia and cardiometabolic traits, with recent studies consistently reporting evidence of partial negative genetic correlation between schizophrenia and body mass index (BMI).^13,14^ However, there is limited evidence for other cardiometabolic and inflammation-related traits,^12^ and the LDSC approach may have limitations. First, there may be a distinction in how common and less common variants are involved in the shared genetic architecture between traits.^15^ This potential difference cannot be captured with LDSC, and so genetic correlation analysis which takes into account the relative frequency of variants is required. Second, LDSC estimates may be biased towards the null when opposing mechanisms exist (eg regions of positive and negative correlation nullifying each other when averaged^16^). This may be expected in a relatively heterogeneous condition like schizophrenia.^17^ For example, a recent study found negligible genetic similarity between schizophrenia and cardiometabolic disorders using LDSC, but evidence in support of a causal relationship when using the same summary GWAS data in MR analysis.^18^ Therefore, more fine-grained locus level genetic correlation analysis is required to identify genomic regions of interest. Third, while LDSC can provide evidence of overall genomic similarity between traits, it cannot provide information with which to consider biological plausibility, or infer potential causality.

Therefore, we used a range of complementary genomic approaches in a stepwise manner to rigorously examine the potential for a common genomic basis for schizophrenia and a number of cardiometabolic and inflammation-related traits previously reported to be associated with it.^19,20^ We aimed to identify specific putative biological pathways underpinning the comorbidity and address key limitations of previous approaches. First, in addition to LDSC to estimate genome-wide correlation of schizophrenia with cardiometabolic and inflammation-related traits, we used genetic covariance analyser (GNOVA^15^), a recent methodological extension of LDSC, to estimate genetic correlation after stratifying variants by minor allele frequency (MAF). Second, we used Heritability Estimation from Summary Statistics (ρ-HESS^16^) to identify positive or negative regions of locus-level genetic correlation that otherwise may be masked by LDSC. Finally, we interrogated genomic regions showing evidence of locus-level correlation for the possibility of common-causal variants in those regions by estimating colocalization between clusters of traits, using the novel hypothesis prioritization multi-trait colocalization (HyPrColoc^21^) method.

## Methods and Materials

### Summary Statistics for Schizophrenia, Cardiometabolic, and Inflammation-related Traits

For schizophrenia, we used publicly available summary data from a recent GWAS from the Psychiatric Genomics Consortium (PGC) (40 675 cases, 64 643 controls^22^). We used publicly available summary GWAS data for twelve cardiometabolic and inflammation-related traits (fasting plasma glucose (FPG), two-hour glucose, fasting insulin (FI), homeostatic model assessment for insulin resistance (HOMA-IR), glycated haemoglobin (HbA1C), type 2 diabetes (T2D), low-density lipoprotein (LDL), high-density lipoprotein (HDL), triglycerides (TG), body mass index (BMI), coronary artery disease (CAD), and CRP) from large-scale consortia (Table 1). All GWAS were conducted in mostly European samples and adjusted for population stratification, age, and sex. Ethical approval was obtained by the original GWAS authors as per each individual GWAS protocol.

**Table 1.** Summary GWAS Statistics Used Cardiometabolic and Inflammatory Traits

Trait	Author, Year (Consortium)	Sample Size	Cases/Controls^a^	Participant Description	PMID
Schizophrenia	Pardinas et al^22^ (PGC)	105318	40 675/64 643	European Adults	29483656
Fasting Insulin	Lagou et al^23^ (MAGIC)	140595	–	European Adults	–
FPG^b^	Scott et al^24^ (MAGIC)	133010	–	European Adults	22885924
HOMA-IR	Dupuis et al^25^ (MAGIC)	46186	–	European Adults	20081858
Two Hour Glucose	Scott et al^24^ (MAGIC)	42854	–	European Adults	22885924
HbA1C	Wheeler et al^26^ (MAGIC)	123665	–	European Adults	28898252
T2DM^b^	Mahajan et al^27^ (DIAGRAM)	898130	74 124/824006	European Adults	30297969
LDL^b^	Liu et al^28^ (GLGC)	237050	–	European Adults	29083408
HDL^b^	Liu et al^28^ (GLGC)	237050	–	European Adults	29083408
Triglycerides^b^	Liu et al^28^ (GLGC)	237050	–	European Adults	29083408
BMI^b^	Pulit et al^29^ (GIANT and UK Biobank)	694649	–	European Adults	30239722
CAD^b^	van der Harst et al^30^ (CARDIoGRAM C4D and UK Biobank)	547261	122733/424528	European Adults	29212778
CRP	Ligthart^31^ (CHARGE)	204402	–	European Adults	30388399

*Note:* FPG, fasting plasma glucose; HOMA, homeostatic model assessment for insulin resistance; HbA1C, glycated haemoglobin; T2DM, type 2 diabetes mellitus; LDL, low-density lipoprotein; HDL, high-density lipoprotein; BMI, body mass index; CAD, coronary artery disease; CRP, C-reactive protein; PGC, psychiatric genomics consortium; MAGIC, meta-analyses of glucose and insulin-related traits consortium; DIAGRAM, diabetes genetics replication and meta-analyses; GLGC, global lipids genetics consortium; GIANT, genetic investigation of anthropometric traits; CARDIoGRAM, coronary artery disease genome wide replication and meta-analysis; C4D, coronary artery disease genetics consortia; CHARGE, cohorts for heart and aging research in genomic epidemiology.

^a^Case/Control numbers supplied for binary traits.

^b^Indicates traits that have previously been paired with schizophrenia and examined for evidence of whole-genome correlation using LDSC.

### Statistical Analysis

#### Linkage Disequilibrium Score Regression for Genome-wide Correlations.

The genome-wide proportion of trait variability explained by shared genetic variation (SNP-heritability; h_2_), and genome-wide correlations (r_g_) between all trait-pairs were estimated using LDSC^32^ and an LD reference panel from the 1000 Genomes Project's Phase 3 European (1kG CEU) sample. Quality control (QC) steps on each GWAS dataset prior to analysis were: (1) filtering SNPs that were not included within the HapMap3 reference panel or had MAF <5% within the 1kG CEU reference sample; (2) filtering SNPs within the major histocompatibility complex (MHC) due to the complex LD structure within the region^33^; filtering out poorly imputed SNPs (INFO > 0.9). Bonferroni adjustment was applied to reduce the risk of type I statistical error due to multiple comparisons. The original alpha level (0.05) was divided by the number of included cardiometabolic and inflammation-related traits which were paired with schizophrenia (12), leaving a Bonferroni-adjusted threshold of *P <* .004 which was used to define strong evidence of genome-wide genetic correlation.

#### MAF-stratified Genetic Correlation.

MAF-stratified genetic correlations between schizophrenia and other traits were estimated using GNOVA.^15^ GNOVA is an extension to classical LDSC, allowing estimates of genetic correlation across continuous annotations (eg MAF). QC methods were the same as above. MAF quartiles were defined by the method authors^15^ and calculated using genotyping data from the 1kG CEU reference sample. MAF cut-offs for each quartile were as follows: Q1 = 0.05–0.11; Q2 = 0.11–0.22; Q3 = 0.22–0.35; Q4 = 0.35–0.50. As per the LDSC analysis described above, the original alpha level (0.05) was divided by the number of included cardiometabolic and inflammation-related traits (12), leaving a Bonferroni-adjusted threshold of *P <* .004 which was used to define strong evidence MAF-stratified genetic correlation. For each trait pair and for each quartile, we report the number of SNPs included in each quartile, correlation estimate, and *P-*value.

#### Locus-level Genetic Correlation.

Next, we explored locus level correlation between schizophrenia and traits with at least nominal evidence of either whole-genome or MAF-stratified genetic correlation. We accepted a less-stringent significance threshold to select traits for locus level correlation analysis to allow for an examination of opposing mechanisms,^16^ which may have biased “averaged” correlation estimates (eg from LDSC or GNOVA) toward the null. We used ρ-HESS^16^ to estimate partitioned heritability and genetic correlations within predefined^34^ genomic LD-blocks based on European participants, allowing for greater resolution of the correlation within each LD block. We assumed no sample overlap between data from different consortia as recommended.^16^ Where no single nucleotide polymorphisms (SNPs) were available for analysis within a particular LD block, that LD block was excluded from analysis. As per the analyses described above, the original alpha level (0.05) divided by the number of traits taken through for locus-level correlation analysis, divided by the number of LD blocks tested between pairs of traits. Therefore, a Bonferroni-adjusted threshold of between *P* = 3.93 × 10^−6^ and 3.38 × 10^−6^ depending on the number of LD blocks per trait pair were used to define strong evidence of locus level genetic correlation.

#### Multi-trait Colocalization.

 To provide greater resolution, allow for a consideration of biological plausibility, triangulate evidence between methods, and examine for a distinction between *correlation vs causation* in genomic regions with evidence of locus-level correlation, we used HyPrColoc.^21^ HyPrColoc estimates the posterior probability of colocalization across multiple traits at a single causal variant by enumerating putative causal configurations. In doing so, HyPrColoc can identify distinct clusters of traits which colocalize at independent putative causal variants within the genomic region of interest. Colocalization analysis does not estimate the direction of association between traits. To conduct this stage of analysis, we identified the lead SNP for schizophrenia within each LD-block showing Bonferroni-significant evidence of locus-level correlation with cardiometabolic and inflammation-related traits (Supplementary Table 1). For each trait, we included all SNPs located 500kb on either side of the schizophrenia lead SNP. We did not consider regions within the MHC. Our primary analysis used the recommended variant-specific prior configuration (prior 1 = 1 × 10^−4^; prior 2 = 0.02) and regional and alignment threshold settings (0.5 for both).

#### Colocalization Sensitivity Analysis.

To test the strength of evidence for colocalization and also cluster stability, we repeated the colocalization analysis over: (1) increasingly stringent prior 2 settings, (0.02, 0.01, 0.001); and (2) increasingly stringent regional and alignment threshold settings (0.5, 0.6, 0.7, 0.8, 0.9). To visualise cluster stability across the permutations, heatmaps were drawn based on a similarity matrix between clusters. Where we found evidence for potential colocalization, stacked regional association plots were drawn to visually inspect putative candidate SNPs, their strength of association within each putative colocalized trait, and the LD structure in the genomic region. Finally, a manual search of the GTEx portal (https://gtexportal.org/home/) was conducted to examine for relevant tissue-specific gene expression of colocalized variants at a Bonferroni-corrected *P-*value threshold based on the number of colocalized SNPs.

## Results

### Genome-wide Correlation between Schizophrenia, Cardiometabolic, and Inflammation-related Traits

Using LDSC, we found Bonferroni-significant evidence of correlation of schizophrenia with BMI (r_g_ = −0.09; 95% C.I., −0.06, −0.12; *P* = 1.83 × 10^−5^; h_2_ = 0.21; SE = 0.007) and T2D (r_g_ = −0.07; 95% C.I., −0.03, −0.12; *P* = .002; h_2_ = 0.04; SE = 0.002). In hierarchical clustering, two clusters were formed; schizophrenia in the first, and all other included traits in the second (Supplementary Figure 1; Supplementary Table 2).

### MAF-stratified Genetic Correlation between Schizophrenia, Cardiometabolic, and Inflammation-related Traits

We found a trend of nominal evidence for correlation in the lowest MAF-quartile between schizophrenia and a range of cardiometabolic and inflammation-related traits (FI (r_g_ = 0.22; *P* = .029); TG (r_g_ = 0.14; *P* = .020); CAD (r_g_ = 0.24; *P* = .025); in the second-lowest MAF-quartile between schizophrenia and LDL (r_g_ = 0.06; *P* = .037); and in the highest MAF-quartile between schizophrenia and both BMI (r_g_ = −0.13; *P* = .006) and T2D (r_g_ = −0.12; *P* = .012) (Supplementary Table 3).

### Locus-level Genetic Correlation between Schizophrenia, Cardiometabolic, and Inflammation-Related Traits

**Fig. 1.**
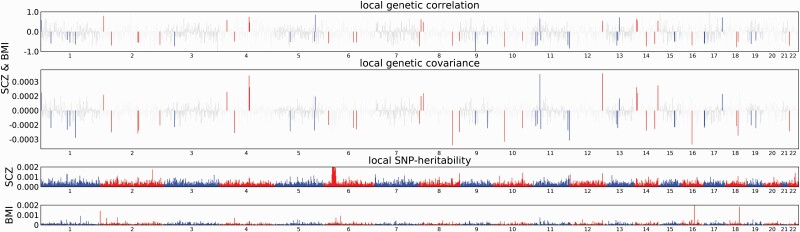
Manhattan plot showing regions of local genetic correlation between schizophrenia and BMI. Manhattan plot showing local genetic correlation estimates (top panel); local covariance estimates (second panel); and local SNP heritability estimates (bottom two panels), at LD blocks across chromosomes 1–22. Areas coloured red/blue in top two panels correspond to LD-blocks surpassing Bonferroni significance threshold. Loci within the major histocompatibility complex (MHC) region were not considered for further analysis due to complex LD patterns in the region. SCZ = schizophrenia; BMI = body mass index.

In total, we identified 85 regions of Bonferroni-significant locus-level correlation between schizophrenia, cardiometabolic, and inflammation-related traits, with *P*-value ranges for the lead schizophrenia SNP of between *P* = 1.11 × 10^−18^ to *P* = 8.80 × 10^−03^. A genome-wide significant SNP for schizophrenia was contained in 24 regions (Supplementary Table 1). All cardiometabolic and inflammation-related traits showed Bonferroni-significant evidence of at least one region of local genetic correlation with schizophrenia. BMI exhibited 62 regions of Bonferroni-significant local genetic correlation with schizophrenia, the most of any trait. Most traits showed evidence of opposing mechanisms with schizophrenia (Table 2; figure 1; Supplementary Table 4; Supplementary Figure 2).

**Table 2.** Summary of Local Genetic Correlation Analyses between Schizophrenia, Cardiometabolic, and Inflammatory Traits

Trait	LD Blocks, No.	Bonferroni *P-*value Threshold^a^	Regions of Local Correlation^b^ with Schizophrenia, No.
BMI	1684	3.38 × 10^−6^	62
Fasting insulin	1676	3.73 × 10^−6^	14
T2D	1591	3.93 × 10^−6^	4
CRP	1684	3.38 × 10^−6^	4
Triglycerides	1684	3.38 × 10^−6^	3
Coronary artery disease	1676	3.73 × 10^−6^	3
HDL	1684	3.38 × 10^−6^	2
LDL	1684	3.38 × 10^−6^	1

*Notes:* BMI, body mass index; T2D, type 2 diabetes mellitus; CRP, C-reactive protein; HDL, high-density lipoprotein; LDL, low-density lipoprotein.

^a^Bonferroni evidential threshold defined as the alpha level (0.05) divided by the number of LD blocks divided by the number of cardiometabolic and inflammation-related traits taken forward for local genetic correlation analysis (8 traits).

^b^Regions identified at Bonferroni-adjusted significance threshold.

### Multi-trait Colocalization between Schizophrenia, Cardiometabolic, and Inflammation-related Traits

**Fig. 2.**
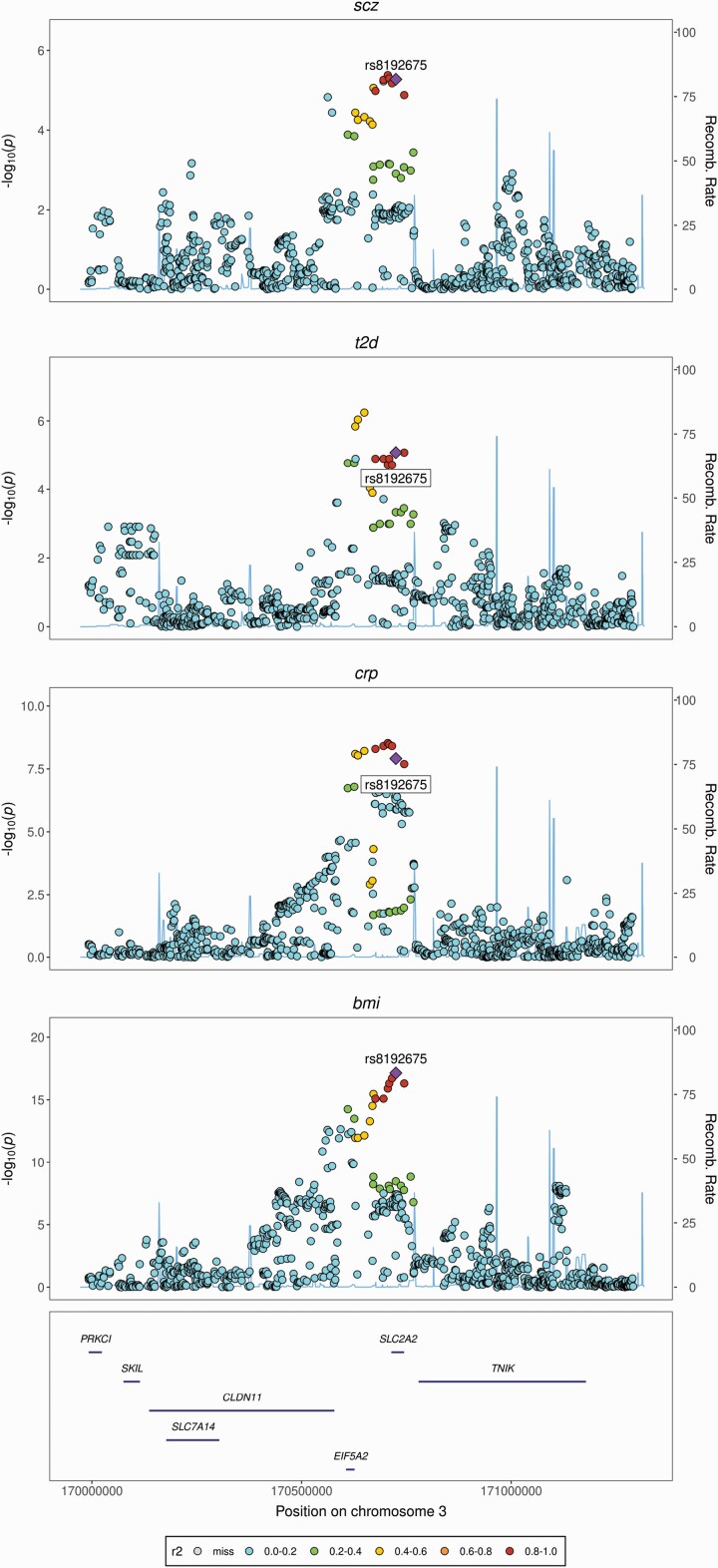

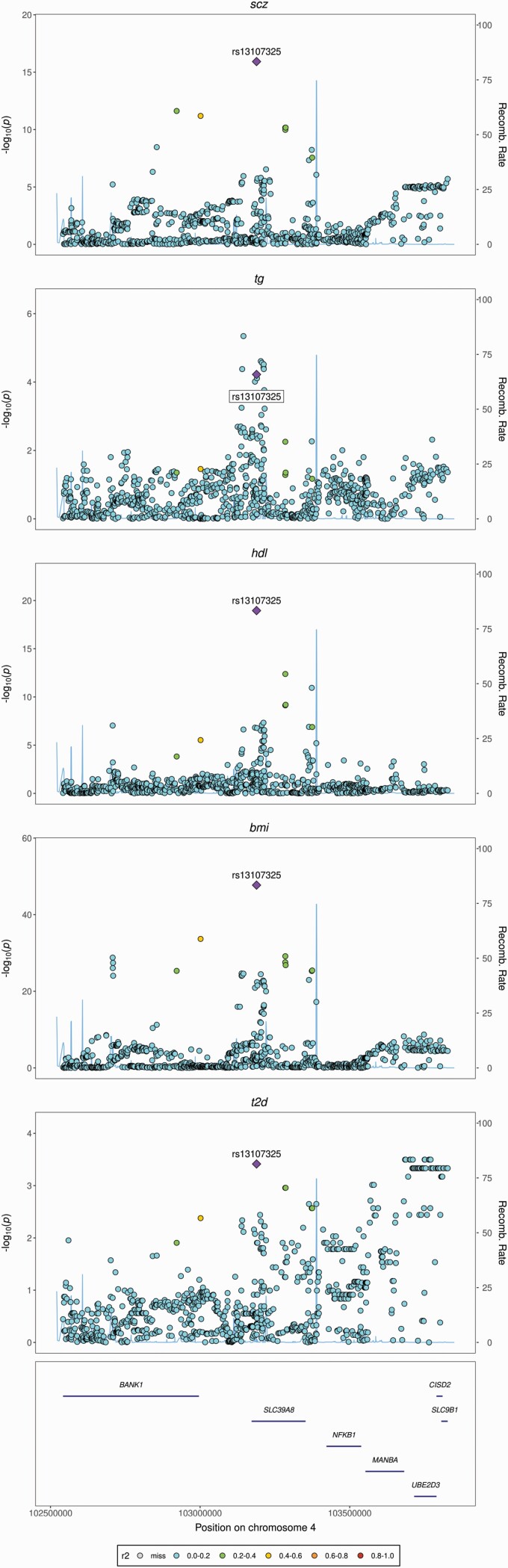

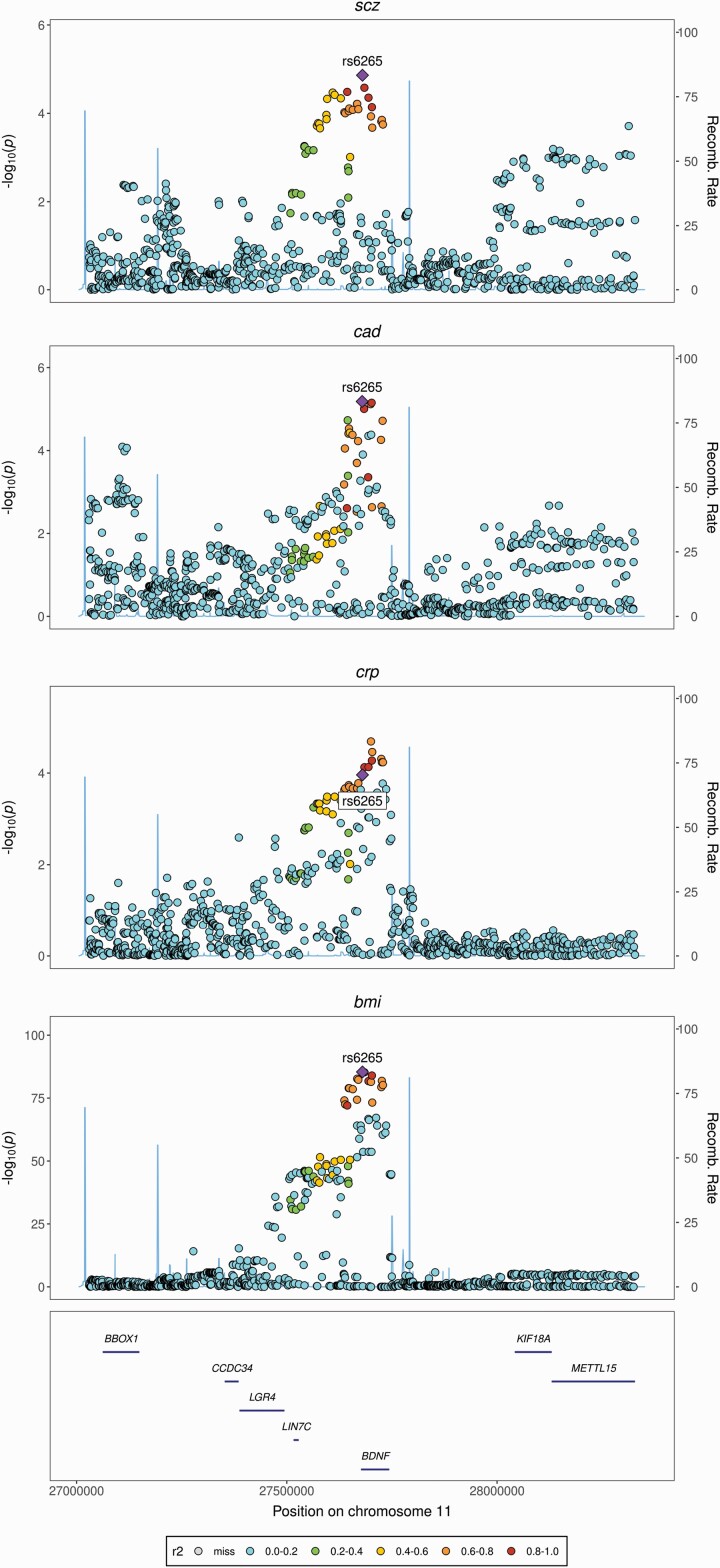

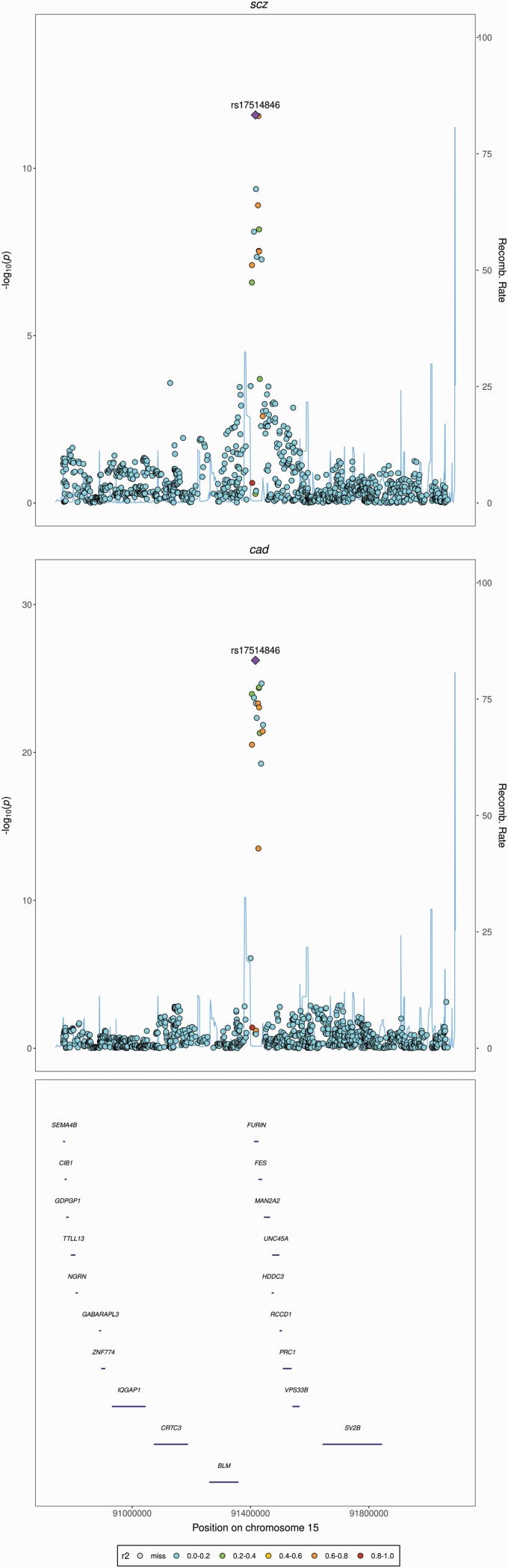
Examples of regional genetic association plots for four loci returning strong evidence for colocalization between schizophrenia, cardiometabolic and inflammatory traits. *A.*rs8192675 – *SLC2A2*, *B.*rs13107325 – *SLC39A8*, *C.*rs6265 – *BDNF*, *D.*rs17514846 - *FURIN*. Regional association plots denote chromosomal location (x axis) and strength of association with listed trait (−log_10(p)_) (y axis) alongside genomic position. SNP r^2^ estimated from the EPIC-Norfolk cohort. See Supplementary Figure 3 for regional association plots of the remaining colocalized variants described in Table 2. Scz = schizophrenia; bmi = body mass index; tg = triglycerides; hdl = high-density lipoprotein; t2ds = type 2 diabetes; crp = c-reactive protein.

We found the strongest evidence for colocalization (posterior probability for colocalization (PP_coloc_) >0.80) between schizophrenia, cardiometabolic, and inflammation-related traits at seven loci, which included missense (rs13107325; rs6265), intronic (rs17514846; rs8192675; rs3800229), and synonymous (rs3814883) variants, and one intergenic variant (rs12782894) (Table 3; figure 2, Supplementary Figure 3). We found additional evidence for colocalization (PP_coloc_ = 0.54–0.79) at three loci, including intronic (rs11191514; rs6031855) and synonymous (rs2239647) variants (Table 3; Supplementary Figure 3). Of the twelve colocalized variants, three (rs17514846; rs13107325; rs6265) are the lead schizophrenia SNPs for the genomic regions, and an additional four (rs3814883; rs8192675; rs3800229; rs2239647) are in strong LD with the lead schizophrenia SNP in the genomic region, based on a European reference population and ascertained using *LDLink* (Table 3). Trait clusters for all loci were stable in sensitivity analysis, returning in all instances the same candidate colocalized variant over increasingly stringent prior and threshold configurations. See Supplementary Table 5 and Supplementary Figure 4 for full sensitivity analysis results and heatmaps. Briefly, clusters at rs17514846 and rs3814883 were stable across all permutations of priors. Clusters at two loci (rs12782894; rs3800229) were stable till prior settings surpassed the most stringent level of 0.99. Clusters at three loci (rs8192675; rs13107325; rs2239647) were stable till regional/alignment thresholds surpassed a stringent level of 0.8, and then T2D was dropped from the clusters and the PP_coloc_ increased for the remaining traits. Clusters at rs6265 were stable till regional/alignment thresholds surpassed 0.7, then CRP was dropped and the PP_coloc_ increased for the remaining traits. Clusters at the remaining two variants (rs11191514; rs6031855) were stable only at the recommended prior settings and regional/alignment thresholds.

**Table 3.** Results from Colocalization Analysis between Schizophrenia, Cardiometabolic, and Inflammatory Traits

Candidate SNP	MAF	Gene Implicated	Variant Type	Colocalized Traits	PP_coloc_^a^	PP_explained_^b^	N SNPs^c^	Relevant Tissue-Specific Gene Expression^d^
rs17514846†	0.461	*FURIN*	Intron	SCZ, CAD, FI	1.00	1.00	1071	Fibroblasts, Pancreas, Whole Blood, Artery, Adipose, Neurone, Heart
rs3814883‡	0.470	*TAOK2*	Synonymous	SCZ, BMI, T2D	0.99	0.99	193	Neurone, Adipose, Whole Blood, Artery, Heart, Thyroid, Fibroblasts, Cerebellum, Pancreas, Lymphocytes, Frontal Cortex, Putamen
rs8192675‡	0.300	*SLC2A2*	Intron	SCZ, BMI, CRP, T2D	0.93	0.50	919	Liver, Pancreas, Whole Blood
rs3800229‡	0.294	*FOXO3*	Intron	SCZ, BMI, CAD	0.89	0.96	872	Neurone, Artery
rs12782894	0.080	^e^	^e^	SCZ, BMI	0.88	0.68	1255	–
rs13107325†	0.077	*SLC39A8*	Missense	SCZ, HDL, TG, BMI, T2D	0.86	1.00	936	Artery, Fibroblasts, Adipose, Lymphocytes, Whole Blood
rs6265†	0.193	*BDNF*	Missense	SCZ, BMI, CRP, CAD, TG, FI	0.86	0.75	925	Artery, Frontal Cortex, Neurone
rs2239647‡	0.457	*AKAP6*	Synonymous	SCZ, BMI, T2D, CAD	0.79	0.66	1584	–
rs11191514	0.087	*CNNM2*	Intron	SCZ, BMI, CAD	0.77	0.30	710	Fibroblasts, Pancreas, Whole Blood, Adipose, Neurone, Lymphocytes, Cerebellum, Artery
rs6031855	0.270	*YWHAB*	Intron	SCZ, BMI	0.59	0.28	990	Whole Blood, Caudate Nucleus, Pancreas, Anterior Cingulate Cortex, Artery, Hypothalamus, Neurone, Nucleus Accumbens, Frontal Cortex, Fibroblasts, Amygdala, Adipose, Heart

*Notes:* SCZ, schizophrenia; BMI, body mass index; CAD, coronary artery disease; HDL, high-density lipoprotein; TG, triglycerides; LDL, low-density lipoprotein; T2D, type 2 diabetes; CRP, C-reactive protein; FI, fasting insulin.

^a^PP_coloc_ indicates posterior probability of single shared causal SNP at default prior and threshold settings.

^b^PP_explained_ indicates the amount of shared trait variance explained by the candidate SNP.

^c^Corresponds to the number of SNPs present in all datasets.

^d^Identified after searching the colocalized SNP on the *GTEx* portal for single-tissue eQTLs at a Bonferroni-corrected threshold of *P* < .004 (for twelve colocalized variants).

^e^Intergenic; †Index SNP for schizophrenia in correlated genomic region; ‡in linkage disequilibrium (LD) with index SNP for schizophrenia in correlated genomic region based on a European reference population *R*^2^ > 0.8; determined via *LDLink*.

## Discussion

Using a complementary set of genomic approaches leveraging GWAS summary data and triangulating the evidence between them, we tested whether schizophrenia and a set of cardiometabolic and inflammation-related traits may share common biologically plausible genetic aetiology. First, we report evidence for partial genome-wide genetic correlation of schizophrenia with T2D and BMI. Second, we report that a “cardiometabolic risk increasing” pattern of partial genetic correlation between schizophrenia and multiple cardiometabolic and inflammation-related traits may be confined to lower-frequency genetic variants within the bounds of GWAS-detectable genetic variation, and a “cardiometabolic protective” pattern of partial genetic correlation may be present amongst the highest-frequency common genetic variants. We identified numerous regions of Bonferroni-significant locus-level genetic correlation between schizophrenia, cardiometabolic, and inflammation-related traits which we interrogated for evidence of common-causal variants using colocalization analysis. In doing so, we found robust and biologically plausible evidence for ten colocalized SNPs that may at least in part contribute toward the comorbidity between schizophrenia, inflammation, and cardiometabolic disorders. Together, our results suggest that the comorbidity between schizophrenia, inflammation, and cardiometabolic disorders could be partly attributable to shared genes, rather than being fully explained by lifestyle factors and medication side-effects.

Findings from our LDSC analysis are in line with previous genomics research. Previous LDSC research has shown a similar negative correlation between schizophrenia and BMI,^13,14^ and a large genotyping meta-analysis has shown an inverse association between polygenic risk for schizophrenia and obesity.^35^ Consistent with this finding, whole-population longitudinal studies have reported that low BMI in childhood is associated with higher risk of schizophrenia in adulthood.^36^ However, our genome-wide SNP-heritability estimate suggests that only a modest fraction of phenotypic variance could be explained by the additive effects of shared genetic variants. This finding from LDSC implies that shared genetic architecture is only likely to partly explain the observational findings.

We also report evidence of a weak Bonferroni-significant negative whole-genome correlation between schizophrenia and T2D, which was not found in a previous LDSC study,^12^ explaining about 4% of phenotypic variance. This is possibly due to the increased power of the more recent GWAS we used in our study. This finding is inconsistent with observational studies suggesting increased T2DM risk in schizophrenia,^4,37^ and studies reporting evidence of positive genetic overlap between schizophrenia and T2DM when using other genomic methods.^38–41^ The observed partial negative correlation between schizophrenia and BMI could be one explanation for this, since T2DM and BMI are themselves highly genetically correlated.^42^ Future research may seek to explore this in more detail. Alternatively, the relatively weak findings from LDSC could be explained by differences in how common and less-common variants are involved in the shared genetic architecture between traits, and/or the presence of opposing mechanisms. Both may downwardly-bias results of whole-genome correlation analysis. Indeed, findings from our MAF-stratified and locus-level correlation analyses support the relevance of the latter interpretation.

Stratifying LDSC by MAF allows an examination of potential differences in how common and less common variants are involved in the shared genetic architecture between traits. We identified a trend of positive genetic correlation between schizophrenia and a range of cardiometabolic and inflammation-related traits in the lowest MAF-quartile, though most results did not surpass the a priori determined Bonferroni significance threshold. This trend of results aligns with observational findings,^19,20^ and also with studies using other methods to examine for genetic overlap between schizophrenia and cardiometabolic traits, eg analyses of GWAS pleiotropy,^40^ polygenic risk score analysis,^38^ transcriptomic and functional enrichment analysis,^41^ and genetic colocalization analysis,^39^ all of which reported evidence in support of genetic similarity between schizophrenia and cardiometabolic traits. We also identified a pattern of “cardioprotective” partial correlation in the highest MAF-quartile of schizophrenia with BMI and T2D, in line with whole-genome correlation estimates. While the flip in effect size for T2D between the lowest and highest MAF quartiles might be due to random allele flips in the original T2D GWAS, the results could also indicate the presence of opposing mechanisms. Nevertheless, the Bonferroni significance threshold was not met for most traits in MAF stratified analysis, and future replication of our work with larger and better-powered GWAS is necessary to confirm these findings.

We found numerous regions of Bonferroni-significant locus-level correlation between included trait-pairs. Amongst the lead schizophrenia SNP within those regions, there was a slight over-representation of SNPs from the lowest MAF-quartile (31 SNPs, 36%). Across trait pairs, we found evidence of multiple regions of positive and negative correlation with schizophrenia, indicating the presence of opposing mechanisms. This may explain the weaker evidence for correlation we found in LDSC and GNOVA, and the results of previous LDSC research^12^ which found limited evidence for genetic correlation between schizophrenia and cardiometabolic traits. This is because the combination of regions of positive and negative correlation may have biased estimates toward the null.^16^

We found ten loci indicating evidence of colocalization between traits at the default prior configuration. Many of these were stable over increasingly stringent settings in sensitivity analysis, suggesting robust evidence for colocalisation, and most are expressed in relevant neurological and cardiometabolic-related tissues. Several loci exhibited stronger evidence for colocalization after one weaker trait was dropped at more stringent thresholds. Of the seven loci returning the strongest evidence of colocalization (PP_coloc_ > 0.80), four (rs6265; rs8192675; rs3800229; rs17514846) relate to pathways involving brain derived neurotrophic factor (BDNF). BDNF is a major member of the neurotrophin family and has been linked with a range of clinical features of schizophrenia^43^; is involved in regulation of cardiometabolic function^44^; and is associated with cardiometabolic indices in people with schizophrenia.^45^

First, rs6265 (Val66Met) is a missense SNP in the *BDNF* gene. Val66Met reduces intracellular trafficking and activity-dependent secretion of BDNF.^46^ Interestingly, meta-analytic evidence suggests lower BDNF levels in people with schizophrenia,^47^ which may contribute to disease-specific changes of both neuronal synaptic plasticity and the immune system.^48^ The Val66Met polymorphism may additionally influence food intake and body weight^49^ in humans.

Second, rs8192675 is located in an intronic region of *SLC2A2*, which encodes the facilitated glucose transporter GLUT2. GLUT2 regulates entry of glucose into the pancreatic β-cell, thus initiating the cascade of events leading to insulin secretion. GLUT2 is also highly expressed in both the liver, where it is involved in the regulation of both glucose uptake and output, and the hypothalamus where it regulates synaptic activity and neurotransmitter release.^50^ Variants in *SLC2A2* impair GLUT2 expression and are strongly associated with T2D.^51^ Rs8192675 is associated with increased diabetic symptomatology but may also be associated with favourable T2D treatment response.^52^ Impaired GLUT2 expression is associated with lower levels of BDNF,^53^ and conversely, higher levels of BDNF are associated with a protective effect on GLUT2 in pancreatic β-cells, reducing T2D risk.^54^

Third, rs3800229 lies in an intron of *FOXO3,* which regulates diverse cellular processes, eg adult stem cell homeostasis^55^ and immuno-metabolic processes.^56^*FOXO3* is associated with brain development and intracranial volume^57^ and is associated with poor cognition in schizophrenia.^58^ Interestingly, *FOXO3* is implicated as a potential therapeutic target for obesity,^59^ and mediates the inhibitory actions of insulin in diverse pathways including cell metabolism and survival.^60^*FOXO3* signalling can be disrupted by BDNF, mediated by the phosphatidylinositol 3-kinase (PI3K)/Akt pathway.^61^ The PI3K/Akt pathway has roles in insulin sensitivity, neuronal development, dopamine regulation, and the immune system,^62^ and has been implicated as a putative mechanism linking schizophrenia and T2D.^63^

Fourth, the rs17514846 variant lies in an intron of *FURIN*, which encodes a protease that processes latent precursor proteins into their biologically active products. *FURIN* is expressed in neuroendocrine, liver, gut, and brain tissues. A recent GWAS found a significant association between rs17514846 and CAD,^64^ and rs17514846 has been shown to regulate *FURIN* expression in monocytes, which modulates their migration and proliferation in atherosclerotic plaques.^65^ Furthermore, rs17514846 is in high-LD with rs4702, a genome-wide significant variant for schizophrenia^66^ which lies in the 3′ untranslated region of *FURIN* leading to reduced gene expression and impaired BDNF secretion.^67^

Outside of BDNF-related pathways, there is biological plausibility for additional colocalized variants. One of these is rs13107325, a missense SNP in *SLC39A8* which encodes a protein responsible for metal ion transport and homeostasis. Rs13107325 has been associated with weight gain,^29^ lipid dysfunction,^68^ changes in brain volume,^69^ and brain metal homeostasis, the latter of which may influence schizophrenia risk.^70^ Finally, two variants, rs3814883 in *TAOK2* and rs11191514 in *CNNM2*, are each associated with schizophrenia,^71,72^ and both are associated with increased risks of cardiometabolic and cardiovascular disorders.^42,73^

The main strengths of our work include the triangulation of evidence from several complementary independent genomic analysis methods that refine genetic correlation estimates to putative common-causal SNPs, permitting a distinction between correlation and causation. In turn, this can inform future basic research, and highlight potential pathways which might be investigated for therapeutic potential for both schizophrenia and its associated cardiometabolic comorbidity. Our findings show a consistent pattern across complimentary methods which are able to address the limitations of previous research. We used stringent Bonferroni correction to reduce the risk of type I statistical error in our work, and combined frequentist analysis with Bayesian colocalization analysis, fostering increased confidence in our findings.

The main limitations of this study are as follows: We considered traits for further analysis based upon a nominal threshold since correlation estimates, which are either (1) averaged across the whole genome (LDSC); or, (2) averaged across MAF quartiles (GNOVA), may have been biased toward the null where opposing mechanisms exist, and, our locus-level correlation analyses suggested this to be the case for schizophrenia with all included traits. Following a priori determined stringent Bonferroni correction, some findings, particularly from MAF-stratified analysis did not reach the evidential threshold. Future better powered analyses may help to confirm our findings. Future work may also consider using other methods to control for the risk of type I statistical error. For our MAF-stratified analysis, due to limitations in current GWAS power, we could only include SNPs with MAF >5% in our lowest MAF-quartile, a limitation common to methods using GWAS data. Such variants are therefore best described as a lower-frequency tranche of common genetic variation. Since we included GWAS data from mostly European samples, it is unclear whether the findings apply to non-European samples. As per the HESS method authors, we assumed no sample overlap for locus-level correlation analysis^16^ owing to the fact that GWAS between analysed trait pairs were conducted by different consortia. Some degree of sample overlap may have been present and this may increase the risk of bias. HyPrColoc assumes the presence of at most one causal SNP in the region, a limitation common to colocalisation methods. Yet, HyPrColoc estimates may only become unreliable when the secondary causal variants explain a similar amount of trait variation as the primary shared variant.^21^ We could only include one inflammatory marker, CRP, since large-scale GWAS of other inflammatory biomarkers are scarce. Despite CRP being a generalized marker of inflammation, future replication of our work with a larger set of upstream inflammatory markers may help to test specific inflammatory pathways. Future research may also consider other mental disorders, eg depression; which is genetically correlated with schizophrenia^74^ and is also observationally associated with cardiometabolic disorders.^75^ Finally, some level of similarity in genetic architecture might be expected between any set of complex disease traits; however, our results show a consistent pattern across a number of analysis methods, suggesting that chance associations are unlikely to fully explain our results.

In conclusion, we present evidence indicating a partial shared genetic basis for schizophrenia, cardiometabolic, and inflammation-related traits, suggesting that the commonly observed comorbidity between these conditions may be partly heritable. Our results imply that LDSC may downwardly bias genetic correlation estimates between schizophrenia, cardiometabolic, and inflammation-related traits. This may be due to the presence of opposing mechanisms, and because the shared genetic aetiology may be confined to relatively lower-frequency common genetic variants. The majority of loci showing evidence for colocalization are biologically plausible, with a number implicating pathways involved in regulation of BDNF. Our results highlight putative pathophysiological mechanisms that could underly the comorbidity, which may form the basis for future basic and therapeutics research, both for schizophrenia and its associated cardiometabolic comorbidity. Future research should also seek to examine the interaction between genetic and environmental factors for the associations of schizophrenia and cardiometabolic disorders.

## Supplementary Material

sgac001_suppl_Supplementary_Data
