# Supplementary material for: Evidence for Shared Genetic Aetiology Between Schizophrenia, Cardiometabolic, and Inflammation-Related Traits: Genetic Correlation and Colocalization Analyses
Source: Schizophr Bull Open. 2022 Jan 11;3(1):sgac001. doi: 10.1093/schizbullopen/sgac001 (PMC8827407; doi:10.1093/schizbullopen/sgac001)
Supplement: sgac001_suppl_Supplementary_Data [file sgac001_suppl_Supplementary_Data.docx]

**Evidence for Genetic Association between Schizophrenia and Cardiometabolic Traits: Correlation and Colocalization Analysis**

*Perry B.I. et al*

**Supplementary Data**

**Supplementary Tables**

**Supplementary Table 1: Lead SNPs for Schizophrenia in Genomic Regions Showing Evidence of Locus Level Correlation between Schizophrenia, Cardiometabolic and Inflammation-related Traits**

| **SNP** | **Chr** | **BP** | ***p*-value for schizophrenia** | **MAF** |
| --- | --- | --- | --- | --- |
| rs11057408 | 12 | 124464836 | 1.55x10^-06^ | 0.266 |
| rs2074754 | 7 | 72891754 | 3.80x10^-03^ | 0.345 |
| rs12911832 | 15 | 58985904 | 8.21x10^-07^ | 0.285 |
| rs6494036 | 15 | 59015961 | 9.68x10^-07^ | 0.291 |
| rs7432375* | 3 | 136288405 | 4.07x10^-12^ | 0.408 |
| rs7257916 | 19 | 45482884 | 1.07x10^-04^ | 0.495 |
| rs115570622 | 3 | 170641344 | 2.18x10^-06^ | 0.231 |
| rs7529073 | 1 | 214147889 | 1.36x10^-06^ | 0.484 |
| rs4274696 | 3 | 124142585 | 1.16x10^-05^ | 0.282 |
| rs2077586* | 2 | 73161551 | 2.96x10^-08^ | 0.299 |
| rs10224497* | 7 | 2149967 | 1.82x10^-15^ | 0.430 |
| rs2332700* | 14 | 72417326 | 1.52x10^-10^ | 0.251 |
| rs2108349 | 7 | 50786663 | 9.04x10^-06^ | 0.702 |
| rs11762176 | 7 | 16562458 | 1.85x10^-05^ | 0.243 |
| rs4650963* | 1 | 177309490 | 1.16x10^-08^ | 0.135 |
| rs10850963 | 12 | 118568002 | 7.98x10^-05^ | 0.433 |
| rs35481364 | 20 | 39764702 | 5.06x10^-05^ | 0.096 |
| rs6771881 | 3 | 38578264 | 2.45x10^-05^ | 0.227 |
| rs13121771 | 4 | 154762293 | 3.56x10^-06^ | 0.483 |
| rs4701099 | 5 | 178777635 | 4.98x10^-05^ | 0.095 |
| rs186209408 | 7 | 68254423 | 8.05x10^-04^ | 0.054 |
| rs11159081 | 14 | 74883356 | 3.09x10^-05^ | 0.500 |
| rs12668848* | 7 | 2020995 | 1.11x10^-18^ | 0.091 |
| rs35297615 | 7 | 127284269 | 2.73x10^-07^ | 0.184 |
| rs9946680 | 18 | 58757452 | 5.16x10^-06^ | 0.068 |
| rs2905627 | 4 | 105450085 | 2.25x10^-07^ | 0.077 |
| rs17514846 | 15 | 91416550 | 2.55x10^-12^ | 0.010 |
| rs6863170 | 5 | 162562220 | 3.65x10^-05^ | 0.076 |
| rs324015 | 12 | 57490100 | 1.42x10^-10^ | 0.241 |
| rs10985817* | 9 | 101071090 | 1.02x10^-09^ | 0.167 |
| rs34685708 | 2 | 162828729 | 3.75x10^-07^ | 0.211 |
| rs17300034 | 7 | 88514733 | 2.60x10^-04^ | 0.350 |
| rs4540690 | 1 | 153825208 | 3.96x10^-06^ | 0.472 |
| rs2480452 | 9 | 131909736 | 2.83x10^-06^ | 0.075 |
| rs13107325* | 4 | 103188709 | 1.19x10^-16^ | 0.071 |
| rs17668001 | 8 | 118557476 | 1.09x10^-07^ | 0.180 |
| rs11646127* | 16 | 29966277 | 5.52x10^-13^ | 0.432 |
| rs893949* | 11 | 134296384 | 2.98x10^-08^ | 0.453 |
| rs117311037 | 10 | 34327891 | 7.55x10^-06^ | 0.100 |
| rs6265 | 11 | 27679916 | 1.36x10^-05^ | 0.193 |
| rs2851447* | 12 | 123665113 | 5.55x10^-16^ | 0.240 |
| rs17024998 | 2 | 40281597 | 8.80x10^-03^ | 0.043 |
| rs10147078 | 14 | 103013178 | 3.74x10^-06^ | 0.375 |
| rs12882110 | 14 | 61680424 | 4.87x10^-06^ | 0.099 |
| rs8137258 | 22 | 20135961 | 9.87x10^-07^ | 0.202 |
| rs5825114* | 18 | 52749216 | 5.02x10^-14^ | 0.251 |
| rs56807175* | 2 | 146436222 | 1.36x10^-11^ | 0.157 |
| rs4837628 | 9 | 122059709 | 1.71x10^-05^ | 0.424 |
| rs17129021 | 14 | 93819544 | 1.75x10^-05^ | 0.697 |
| rs56007784 | 17 | 1290950 | 1.48x10^-06^ | 0.275 |
| rs1518002 | 4 | 18843657 | 1.66x10^-06^ | 0.396 |
| rs10754948 | 2 | 15305738 | 2.06x10^-06^ | 0.261 |
| rs500102 | 9 | 77358745 | 3.41x10^-07^ | 0.405 |
| rs35662245 | 2 | 147583187 | 4.34x10^-07^ | 0.171 |
| rs7701440* | 5 | 60620980 | 3.72x10^-14^ | 0.491 |
| rs13170232* | 5 | 152899532 | 1.23x10^-08^ | 0.098 |
| rs1044433 | 17 | 72947061 | 1.19x10^-05^ | 0.311 |
| rs12652777* | 5 | 155775075 | 8.32x10^-08^ | 0.500 |
| rs9398172 | 6 | 108994826 | 1.38x10^-06^ | 0.294 |
| rs12142033 | 1 | 39542129 | 3.37x10^-06^ | 0.335 |
| rs10603579 | 11 | 13032679 | 1.08x10^-03^ | 0.050 |
| rs4648845* | 1 | 2387101 | 6.74x10^-12^ | 0.094 |
| rs58033671* | 8 | 143320118 | 8.65x10^-18^ | 0.062 |
| rs34179565* | 14 | 33298731 | 8.88x10^-09^ | 0.393 |
| rs61373214 | 10 | 104313527 | 1.05x10^-10^ | 0.094 |
| rs117178675 | 19 | 13629414 | 9.20x10^-06^ | 0.079 |
| rs35432584 | 13 | 56834975 | 3.71x10^-06^ | 0.422 |
| rs9810544 | 3 | 31534464 | 9.13x10^-07^ | 0.131 |
| rs4627212 | 13 | 58822051 | 3.47x10^-07^ | 0.302 |
| rs979618 | 5 | 165846092 | 7.64x10^-04^ | 0.053 |
| rs6960644 | 7 | 12569268 | 4.87x10^-04^ | 0.175 |
| rs2567363 | 3 | 197224294 | 4.02x10^-05^ | 0.251 |
| rs4722797 | 7 | 28484650 | 1.21x10^-06^ | 0.243 |
| rs67712855 | 20 | 43682551 | 3.32x10^-06^ | 0.317 |
| rs330745 | 1 | 187232765 | 4.44x10^-05^ | 0.069 |
| rs79367205 | 1 | 103272071 | 1.03x10^-04^ | 0.082 |
| rs277245 | 12 | 43999677 | 6.38x10^-05^ | 0.233 |
| rs28648547 | 7 | 129734280 | 2.43x10^-05^ | 0.405 |
| rs2545291 | 4 | 181647829 | 7.43x10^-04^ | 0.068 |
| rs12148492 | 15 | 51653193 | 4.94x10^-05^ | 0.182 |
| rs143360647 | 17 | 42857175 | 5.86x10^-05^ | 0.033 |
| rs495269 | 6 | 145402340 | 5.50x10^-03^ | 0.688 |
| rs35662245 | 2 | 147583187 | 5.50x10^-03^ | 0.171 |
| rs10968565 | 9 | 28405415 | 4.34x10^-07^ | 0.059 |
| rs17668001 | 8 | 118557476 | 1.09x10^-07^ | 0.190 |
| rs28712486 | 9 | 86481873 | 1.82x10^-05^ | 0.118 |
| rs1995357 | 12 | 117945315 | 6.09x10^-07^ | 0.598 |
| rs73224901 | 4 | 29405992 | 5.60x10^-04^ | 0.031 |
| rs11041236 | 11 | 7216013 | 1.88x10^-04^ | 0.020 |
| rs3120864 | 13 | 55737401 | 1.30x10^-03^ | 0.165 |
| rs56007784 | 17 | 1290950 | 1.48x10^-06^ | 0.313 |
| rs7596038* | 2 | 58383820 | 2.37x10^-12^ | 0.447 |
| rs140130327 | 3 | 191341579 | 7.00x10^-07^ | 0.013 |
| rs12066151 | 1 | 74465975 | 7.06x10^-05^ | 0.142 |
| rs116767569 | 3 | 181969815 | 1.76x10^-04^ | 0.022 |
| rs28627277 | 17 | 47042840 | 5.43x10^-06^ | 0.138 |
| rs16902086* | 5 | 45285752 | 5.55x10^-11^ | 0.352 |
| rs55875953 | 18 | 42301842 | 5.65x10^-04^ | 0.191 |
| rs140713283 | 10 | 90465038 | 1.13x10^-04^ | 0.010 |

*indicates genome-wide significant SNP for schizophrenia

**Supplementary Table 2: LDSC Results for Schizophrenia, Cardiometabolic and Inflammatory Traits**

| **Trait 1** | **Trait 2** | **RG** | **SE** | **Z-Score** | **H_2_** | **H_2_ SE** | ***p*-value** |
| --- | --- | --- | --- | --- | --- | --- | --- |
| Schizophrenia | Fasting Insulin | -0.0285 | 0.038 | -0.733 | 0.079 | 0.008 | 0.463 |
| Schizophrenia | HDL | -0.074 | 0.032 | 1.412 | 0.127 | 0.024 | 0.158 |
| Schizophrenia | Triglycerides | 0.035 | 0.029 | -0.858 | 0.168 | 0.030 | 0.391 |
| Schizophrenia | LDL | -0.023 | 0.022 | -0.977 | 0.201 | 0.049 | 0.329 |
| Schizophrenia | FPG | -0.048 | 0.037 | -1.311 | 0.075 | 0.015 | 0.190 |
| Schizophrenia | BMI | -0.091 | 0.015 | -5.924 | 0.205 | 0.007 | <0.001 |
| Schizophrenia | T2D | -0.073 | 0.023 | -3.14 | 0.043 | 0.002 | 0.002 |
| Schizophrenia | Two Hour Glucose | -0.020 | 0.062 | -0.33 | 0.030 | 0.010 | 0.743 |
| Schizophrenia | HOMA-IR | -0.028 | 0.050 | -0.56 | 0.038 | 0.007 | 0.578 |
| Schizophrenia | HbA1C | -0.009 | 0.029 | -0.29 | 0.074 | 0.009 | 0.771 |
| Schizophrenia | CAD | 0.029 | 0.019 | 1.52 | 0.108 | 0.008 | 0.129 |
| Schizophrenia | CRP | -0.017 | 0.003 | -0.635 | 0.232 | 0.009 | 0.205 |
| Two Hour Glucose | T2DM | 0.400 | 0.098 | 4.06 | 0.041 | 0.003 | 0.002 |
| Two Hour Glucose | HOMA-IR | -0.094 | 0.179 | -0.527 | 0.039 | 0.007 | 0.598 |
| Two Hour Glucose | HbA1C | 0.371 | 0.115 | 3.24 | 0.077 | 0.009 | 0.001 |
| Two Hour Glucose | CAD | 0.258 | 0.088 | 2.95 | 0.064 | 0.005 | 0.003 |
| Two Hour Glucose | LDL | -0.001 | 0.078 | -0.013 | 0.207 | 0.048 | 0.986 |
| Two Hour Glucose | HDL | 0.101 | 0.104 | 0.968 | 0.112 | 0.020 | 0.333 |
| Two Hour Glucose | Fasting Insulin | 0.057 | 0.119 | 0.476 | 0.083 | 0.009 | 0.640 |
| Two Hour Glucose | FPG | 0.027 | 0.156 | 0.172 | 0.075 | 0.016 | 0.863 |
| Two Hour Glucose | BMI | -0.038 | 0.054 | -0.711 | 0.203 | 0.006 | 0.477 |
| Two Hour Glucose | CRP | 0.026 | 0.096 | 0.271 | 0.087 | 0.019 | 0.786 |
| Two Hour Glucose | Triglycerides | 0.081 | 0.098 | 0.823 | 0.144 | 0.025 | 0.410 |
| HOMA-IR | T2D | 0.624 | 0.088 | 7.11 | 0.042 | 0.003 | <0.001 |
| HOMA-IR | HbA1C | 0.195 | 0.081 | 2.40 | 0.078 | 0.009 | 0.016 |
| HOMA-IR | CAD | 0.222 | 0.071 | 3.12 | 0.065 | 0.005 | 0.002 |
| HOMA-IR | LDL | 0,021 | 0.056 | 0.357 | 0.209 | 0.045 | 0.722 |
| HOMA-IR | HDL | -0.597 | 0.098 | -6.122 | 0.112 | 0.019 | <0.001 |
| HOMA-IR | Fasting Insulin | 1.000 | 0.079 | 15.078 | 0.037 | 0.008 | <0.001 |
| HOMA-IR | FPG | 0.354 | 0.093 | 3.810 | 0.075 | 0.016 | <0.001 |
| HOMA-IR | BMI | 0.667 | 0.075 | 8.844 | 0.304 | 0.063 | <0.001 |
| HOMA-IR | CRP | 0.447 | 0.124 | 3.603 | 0.088 | 0.018 | <0.001 |
| HOMA-IR | Triglycerides | 0.527 | 0.100 | 5.266 | 0.141 | 0.024 | <0.001 |
| T2D | HbA1C | 0.466 | 0.044 | 10.66 | 0.075 | 0.009 | <0.001 |
| T2D | CAD | 0.389 | 0.034 | 11.58 | 0.065 | 0.005 | <0.001 |
| T2D | LDL | 0.063 | 0.041 | 1.531 | 0.201 | 0.049 | 0.126 |
| T2D | HDL | -0.415 | 0.038 | -11.05 | 0.109 | 0.014 | <0.001 |
| T2D | Fasting Insulin | 0.556 | 0.056 | 10.00 | 0.041 | 0.003 | <0.001 |
| T2D | FPG | 0.471 | 0.074 | 6.398 | 0.076 | 0.016 | <0.001 |
| T2D | BMI | 0.554 | 0.028 | 19.735 | 0.205 | 0.007 | <0.001 |
| T2D | CRP | 0.349 | 0.056 | 5.899 | 0.018 | 1.022 | <0.001 |
| T2D | Triglycerides | 0.392 | 0.057 | 6.82 | 0.131 | 0.022 | <0.001 |
| CAD | HbA1C | 0.253 | 0.035 | 7.234 | 0.075 | 0.009 | <0.001 |
| CAD | LDL | 0.189 | 0.045 | 4.174 | 0.201 | 0.045 | <0.001 |
| CAD | HDL | -0.316 | 0.033 | -0.696 | 0.108 | 0.016 | <0.001 |
| CAD | Fasting Insulin | 0.305 | 0.050 | 6.145 | 0.107 | 0.008 | <0.001 |
| CAD | FPG | 0.121 | 0.042 | 2.892 | 0.076 | 0.016 | 0.004 |
| CAD | BMI | 0.307 | 0.021 | 14.675 | 0.205 | 0.007 | <0.001 |
| CAD | CRP | 0.242 | 0.060 | 4.050 | 0.089 | 0.018 | <0.001 |
| CAD | Triglycerides | 0.283 | 0.032 | 6.925 | 0.131 | 0.023 | <0.001 |
| HbA1C | LDL | 0.152 | 0.057 | 2.682 | 0.199 | 0.044 | 0.0073 |
| HbA1C | HDL | -0.131 | 0.049 | -2.670 | 0.105 | 0.018 | 0.0076 |
| HbA1C | Fasting Insulin | 0.182 | 0.068 | 2.647 | 0.076 | 0.009 | 0.008 |
| HbA1C | FPG | 0.522 | 0.084 | 6.235 | 0.076 | 0.016 | <0.001 |
| HbA1C | BMI | 0.237 | 0.028 | 8.571 | 0.204 | 0.007 | <0.001 |
| HbA1C | CRP | 0.225 | 0.056 | 4.064 | 0.087 | 0.019 | <0.001 |
| HbA1C | Triglycerides | 0.167 | 0.054 | 3.082 | 0.133 | 0.024 | 0.0021 |
| CRP | Fasting Insulin | 0.353 | 0.087 | 4.085 | 0.088 | 0.019 | <0.001 |
| CRP | HDL | -0.325 | 0.062 | -5.289 | 0.127 | 0.023 | <0.001 |
| CRP | Triglycerides | 0.298 | 0.093 | 3.202 | 0.169 | 0.029 | 0.001 |
| CRP | LDL | 0.157 | 0.102 | 1.544 | 0.202 | 0.050 | 0.123 |
| CRP | FPG | 0.230 | 0.080 | 2.888 | 0.076 | 0.015 | 0.004 |
| CRP | BMI | 0.480 | 0.056 | 8.660 | 0.204 | 0.006 | <0.001 |
| Triglycerides | Fasting Insulin | 0.415 | 0.099 | 4.211 | 0.138 | 0.025 | <0.001 |
| Triglycerides | HDL | -0.568 | 0.063 | -9.007 | 0.131 | 0.023 | <0.001 |
| Triglycerides | LDL | 0.381 | 0.053 | 7.15 | 0.201 | 0.047 | <0.001 |
| Triglycerides | FPG | 0.144 | 0.092 | 1.490 | 0.075 | 0.016 | 0.139 |
| Triglycerides | BMI | 0.281 | 0.037 | 7.700 | 0.204 | 0.007 | <0.001 |
| HDL | LDL | -0.018 | 0.065 | -0.272 | 0.209 | 0.047 | 0.786 |
| HDL | FPG | -0.209 | 0.076 | -2.759 | 0.075 | 0.016 | 0.006 |
| HDL | BMI | -0.396 | 0.036 | -11.034 | 0.204 | 0.007 | <0.001 |
| HDL | Fasting Insulin | -0.537 | 0.078 | -6.908 | 0.104 | 0.021 | <0.001 |
| Fasting Insulin | LDL | 0.079 | 0.054 | 1.480 | 0.212 | 0.047 | 0.139 |
| Fasting Insulin | FPG | 0.327 | 0.104 | 3.148 | 0.074 | 0.016 | 0.001 |
| Fasting Insulin | BMI | 0.587 | 0.043 | 13.690 | 0.204 | 0.007 | <0.001 |

HDL=high-density lipoprotein; LDL=low-density lipoprotein; FPG=fasting plasma glucose; BMI=body mass index; T2D=type 2 diabetes mellitus; HOMA=homeostatic model assessment of insulin resistance; HbA1C=glycated haemoglobin; CAD=coronary artery disease; CRP=C-reactive protein.

| **Trait** | **MAF^a^** | | | | | | | | | | | |
| --- | --- | --- | --- | --- | --- | --- | --- | --- | --- | --- | --- | --- |
|  | **Q1** | | | **Q2** | | | **Q3** | | | **Q4** | | |
|  | ***n* SNPs^b^** | **r_g_** | ***p*** | ***n* SNPs^b^** | **r_g_** | ***p*** | ***n* SNPs^b^** | **r_g_** | ***p*** | ***n* SNPs^b^** | **r_g_** | ***p*** |
| T2D^†^ | 181456/1046803 | 0.062 | 0.076 | 256776/1046803 | 0.045 | 0.585 | 297430/1046803 | -0.056 | 0.433 | 311141/1046803 | -0.120 | 0.012 |
| FPG | 183141/1055741 | 0.078 | 0.296 | 259122/1055741 | 0.052 | 0.971 | 299926/1055741 | 0.143 | 0.345 | 313552/1055741 | -0.127 | 0.173 |
| Fasting Insulin^†^ | 169246/976878 | 0.223 | 0.029 | 241316/976878 | 0.110 | 0.266 | 277470/976878 | 0.095 | 0.174 | 288846/976878 | -0.050 | 0.790 |
| HOMA-IR | 167969/959775 | -0.122 | 0.507 | 237685/959775 | -0.147 | 0.284 | 271834/959775 | 0.056 | 0.652 | 282287/959775 | -0.067 | 0.570 |
| Two Hour Glucose | 164391/936379 | 0.030 | 0.220 | 232596/936379 | -0.112 | 0.670 | 264999/936379 | -0.063 | 0.691 | 274393/936379 | 0.023 | 0.842 |
| HbA1C | 172506/999766 | 0.013 | 0.459 | 246096/999766 | 0.032 | 0.844 | 282284/999766 | -0.097 | 0.350 | 296910/999766 | 0.044 | 0.414 |
| HDL^†^ | 164130/937257 | -0.114 | 0.053 | 232995/937257 | -0.070 | 0.280 | 265389/937257 | 0.043 | 0.105 | 274743/937257 | 0.051 | 0.572 |
| LDL^†^ | 163864/936663 | 0.053 | 0.371 | 232851/936663 | 0.063 | 0.037 | 265279/936663 | 0.036 | 0.291 | 274669/936663 | 0.039 | 0.231 |
| Triglycerides^†^ | 163905/936714 | 0.136 | 0.020 | 232870/936714 | 0.025 | 0.106 | 265280/936714 | -0.003 | 0.188 | 274659/936714 | 0.031 | 0.201 |
| BMI^†^ | 170187/971687 | -0.109 | 0.219 | 240402/971687 | -0.086 | 0.187 | 275135/971687 | -0.078 | 0.143 | 285963/971687 | -0.127 | 0.006 |
| CAD ^†^ | 182837/1054110 | 0.235 | 0.025 | 258637/1054110 | -0.025 | 0.761 | 299487/1054110 | 0.054 | 0.484 | 313149/1054110 | -0.041 | 0.446 |
| CRP^†^ | 177187/1018650 | 0.181 | 0.088 | 250474/1018650 | 0.018 | 0.859 | 289207/1018650 | 0.091 | 0.196 | 301782/1018650 | -0.049 | 0.358 |

**Supplementary Table 3: MAF Stratified Genetic Correlations between Schizophrenia, Cardiometabolic and Inflammatory Traits**

HDL=high-density lipoprotein; LDL=low-density lipoprotein; FPG=fasting plasma glucose; BMI=body mass index; T2D=type 2 diabetes mellitus; HOMA=homeostatic model assessment of insulin resistance; HbA1C=glycated haemoglobin; CAD=coronary artery disease; CRP=C-reactive protein; r_g_=genetic correlation estimate
^a^MAF split into quartiles; Q1=lowest to Q4=highest.

^b^The first number corresponds to the number of SNPs for schizophrenia. The second number corresponds to the number of SNPs for the trait listed in the ‘Trait’ column.
^†^indicates traits taken to next stage of analysis based upon nominal evidence of whole or stratified genetic correlation.

**Supplementary Table 4: Regions of Local Genetic Correlation Surpassing Bonferroni Evidential Threshold between Schizophrenia and Cardiometabolic and Inflammatory Traits**

| **Chromosome** | **Start** | **End** | **Local Genetic Correlation**  **(95% CI)** | ***p-value*** |
| --- | --- | --- | --- | --- |
| **Fasting Insulin** | | | | |
| 5 | 165642395 | 166847740 | 0.92 (0.66-0.97) | 2.01E-12 |
| 7 | 11299198 | 12635461 | 1.00 (0.65-1.00) | 5.99E-11 |
| 3 | 197075987 | 197946622 | 0.73 (0.42-0.98) | 5.24E-09 |
| 7 | 28360309 | 31137289 | 0.70 (0.42-0.96) | 1.47E-07 |
| 20 | 42680176 | 44839056 | 0.66 (0.41-0.87) | 2.71E-07 |
| 1 | 186810023 | 188759945 | 0.62 (0.31-0.87) | 4.87E-07 |
| 1 | 102898745 | 103914211 | 0.66 (0.30-0.92) | 5.60E-07 |
| 12 | 43984474 | 46024229 | 0.62 (0.42-0.87) | 5.92E-07 |
| 7 | 128778386 | 130422414 | -0.90 (-1.00 - -0.54) | 5.96E-07 |
| 4 | 180122043 | 182066807 | 1.00 (0.67-1.00) | 8.76E-07 |
| 15 | 50008043 | 51677560 | 0.63 (0.37-0.77) | 1.28E-06 |
| 17 | 41772087 | 43056905 | -0.82 (-1.00 - -0.49) | 1.71E-06 |
| 6 | 145319810 | 146665424 | -0.81 (-1.00 - -0.38) | 2.31E-06 |
| 2 | 147277162 | 150210292 | -0.78 (-1.00 - -0.51) | 2.43E-06 |
| **Type 2 Diabetes Mellitus** | | | | |
| 4 | 154477641 | 155056126 | 0.49 (0.29-0.76) | 3.2670e-06 |
| 5 | 178413464 | 179401244 | 0.45 (0.25-0.76) | 5.9955e-06 |
| 7 | 68234074 | 69085364 | 0.66 (0.37-0.96) | 7.1611e-06 |
| 14 | 72889615 | 76444767 | 0.42 (0.42-0.64) | 7.8567e-06 |
| **High-Density Lipoprotein** | | | | |
| 6 | 30798168 | 31571218 | 0.56 (0.37-0.75) | 9.3891e-09 |
| 12 | 122007651 | 124977980 | -0.79 (-1.00 - -0.50) | 1.4944e-07 |
| **Low-Density Lipoprotein** | | | | |
| 6 | 31571218 | 32682664 | 0.52 (0.32-0.72) | 4.47e-07 |
| **Triglycerides** | | | | |
| 12 | 122007651 | 124977980 | 0.57 (0.37-0.78) | 2.9534e-08 |
| 15 | 58441366 | 59694116 | 0.350 (0.22-0.49) | 8.1201e-07 |
| 10 | 100668400 | 102949239 | -0.77 (-1.00 - -0.58) | 4.4472e-06 |
| **Body Mass Index** | | | | |
| 4 | 100678360 | 103221356 | 0.74 (0.58-1.00) | 1.8800e-18 |
| 8 | 116096495 | 119685457 | -0.71 (-0.87- -0.64) | 1.5271e-17 |
| 16 | 29036613 | 31382943 | -0.69 (-0.85- -0.42) | 3.9790e-17 |
| 11 | 134205993 | 134946452 | -0.85 (-1.00 - -0.44) | 7.6067e-17 |
| 10 | 33707968 | 35109355 | -0.76 (-0.95 - -0.60) | 1.6559e-16 |
| 11 | 27020461 | 28481593 | 0.65 (0.49-0.70) | 1.3360e-15 |
| 12 | 122007651 | 124977980 | 0.54 (0.41-1.00) | 3.0322e-15 |
| 1 | 154770403 | 156336133 | -0.64 (-0.81 - -0.44) | 2.8648e-13 |
| 14 | 29972145 | 32383265 | 0.65 (0.45-1.00) | 1.0969e-10 |
| 2 | 40281483 | 43309590 | -0.69 (-0.90 - -0.62) | 1.5763e-10 |
| 14 | 103012102 | 105001723 | 0.55 (0.38-1.00) | 2.6666e-10 |
| 14 | 61680424 | 63790015 | -0.74 (-0.97- -0.68) | 4.7168e-10 |
| 22 | 19912358 | 22357325 | -0.70 (-0.93 - -0.59) | 7.4182e-10 |
| 18 | 51554175 | 55213838 | -0.52 (-0.68 - -0.33) | 9.0991e-10 |
| 2 | 144519484 | 146445570 | -0.55 (-0.73 - -0.43) | 2.0740e-09 |
| 11 | 130342575 | 131074612 | -0.68 (-0.90 - -0.59) | 2.6699e-09 |
| 4 | 103221356 | 105305294 | 0.48 (0.32-0.94) | 8.0355e-09 |
| 9 | 121321537 | 122260297 | -0.63 (-0.85- -0.51) | 2.5874e-08 |
| 14 | 93132299 | 94325285 | -0.61 (-0.83 - -0.54) | 2.7201e-08 |
| 17 | 1172399 | 1928731 | -0.62 (-0.84 - -0.49) | 2.8907e-08 |
| 4 | 18841874 | 20544557 | 0.58 (0.38-1.00) | 3.4517e-08 |
| 2 | 14335308 | 16329735 | 0.80 (0.51-1.00) | 4.4638e-08 |
| 9 | 76973081 | 78900183 | -1.00 (-1.00- -1.00) | 4.7885e-08 |
| 2 | 147277162 | 150210292 | -0.55 (-0.75-0.43) | 4.8358e-08 |
| 5 | 58524622 | 60935907 | -0.53 (-0.72 - -0.38) | 5.0203e-08 |
| 5 | 152867774 | 153773088 | -0.50 (-0.68 - -0.43) | 5.8424e-08 |
| 17 | 72672203 | 74375560 | 0.71 (0.45-1.00) | 6.5898e-08 |
| 5 | 155373505 | 156628700 | 0.85 (0.54-1.00) | 7.4102e-08 |
| 6 | 108464380 | 110304247 | -0.54 (-0.73 - -0.43) | 9.8576e-08 |
| 1 | 38731847 | 40200567 | -0.59 (-0.81 - -0.43) | 1.2270e-07 |
| 11 | 12564229 | 13373124 | -0.73 (-0.99 - -0.52) | 1.2379e-07 |
| 18 | 45939732 | 47730584 | -0.78 (-1.00 - -0.60) | 1.7993e-07 |
| 4 | 43965045 | 45189157 | -0.49 (-0.69 - -0.22) | 2.0291e-07 |
| 1 | 1892607 | 3582736 | 0.58 (0.36-1.00) | 2.0822e-07 |
| 8 | 143044914 | 144236881 | -0.46 (-0.64 - -0.72) | 2.1199e-07 |
| 14 | 32383265 | 34846251 | 0.56 (0.34-1.00) | 2.8869e-07 |
| 10 | 102949239 | 104380410 | -0.46 (-0.64- -0.40) | 4.2282e-07 |
| 19 | 13471127 | 14486347 | -0.62 (-0.85 - -0.73) | 4.2562e-07 |
| 13 | 55817131 | 57554217 | -0.70 (-0.97 - -0.45) | 4.6785e-07 |
| 3 | 30717955 | 32351715 | -0.73 (-1.00 - -0.42) | 5.0177e-07 |
| 13 | 58410626 | 59302271 | -0.45 (-0.63- -0.38) | 6.9698e-07 |
| 8 | 143044914 | 144236881 | -0.46 (-0.64 - -0.32) | 2.1199e-07 |
| 14 | 32383265 | 34846251 | 0.56 (0.35-1.00) | 2.8869e-07 |
| 10 | 102949239 | 104380410 | -0.46 (-0.64- -0.50) | 4.2282e-07 |
| 19 | 13471127 | 14486347 | -0.62 (-0.85 - -0.73) | 4.2562e-07 |
| 13 | 55817131 | 57554217 | -0.70 (-0.98 - -0.55) | 4.6785e-07 |
| 3 | 30717955 | 32351715 | -0.73 (-1.00 - -0.54) | 5.0177e-07 |
| 13 | 58410626 | 59302271 | -0.44 (-0.65 - -0.38) | 6.9698e-07 |
| 2 | 229370787 | 231843389 | -0.66 (-0.93 - -0.48) | 1.0193e-06 |
| 8 | 9640787 | 10463197 | 0.49 (0.29-0.94) | 1.3453e-06 |
| 6 | 97842284 | 100630146 | -0.54 (-0.76 - -0.31) | 1.3989e-06 |
| 13 | 63971559 | 65200602 | 0.72 (0.43-1.00) | 1.4903e-06 |
| 15 | 76398624 | 78516053 | -0.52 (-0.73 - -0.42) | 1.5391e-06 |
| 11 | 30141357 | 32276901 | -0.44 (-0.62 - -0.38) | 1.5419e-06 |
| 17 | 1928731 | 3702312 | -0.57 (-0.81 - -0.39) | 1.8773e-06 |
| 19 | 30727954 | 32746520 | -0.45 (-0.64 - -0.35) | 1.9589e-06 |
| 8 | 4480476 | 5146927 | 0.62 (0.36-1.71) | 1.9798e-06 |
| 11 | 17578402 | 19569535 | -0.68 (-0.96- -0.81) | 2.8624e-06 |
| 6 | 13209388 | 14802924 | -0.59 (-0.84 – 0.43) | 3.0978e-06 |
| 1 | 106087842 | 108409665 | -0.55 (-0.78 - -0.33) | 3.2491e-06 |
| 8 | 2573279 | 3392926 | -0.74 (-1.00 - -0.44) | 3.3313e-06 |
| **Coronary Artery Disease** | | | | |
| 10 | 104380410 | 106695048 | 0.47 (0.33-0.77) | 1.8131e-12 |
| 15 | 90475551 | 92164392 | -0.55 (-0.72 - -0.29) | 3.2537e-10 |
| 7 | 128778386 | 130422414 | 0.64 (0.39-0.66) | 8.4244e-07 |
| **C-Reactive Protein** | | | | |
| 7 | 87825004 | 90661784 | 0.99 (0.88-1.00) | 2.3160e-08 |
| 1 | 153180829 | 154770403 | -0.40 (-0.56- -0.11) | 7.0758e-07 |
| 9 | 130055510 | 132165470 | -0.96 (-1.00 - -0.90) | 3.8599e-06 |
| 2 | 60292000 | 62429044 | -0.87 (-1.00 - -0.66) | 1.2429e-05 |

**Supplementary Table 5: Sensitivity Analysis Results for Schizophrenia, Cardiometabolic and Inflammatory Traits: Modifying Prior Configurations and Regional/Alignment Thresholds**

| **Candidate SNP** | **Colocalized Traits** | **PP_coloc_^1^** | **PP_explained_^2^** | **N SNPs^3^** | **Prior Prob 2** | **Reg/Align**  **Threshold** |
| --- | --- | --- | --- | --- | --- | --- |
| rs8192675 | SCZ, T2D, CRP, BMI | 0.9299 | 0.5033 | 919 | 0.95 | 0.5 |
| rs8192675 | SCZ, T2D, CRP, BMI | 0.9299 | 0.5033 | 919 | 0.95 | 0.6 |
| rs8192675 | SCZ, T2D, CRP, BMI | 0.9299 | 0.5033 | 919 | 0.95 | 0.7 |
| rs8192675 | SCZ, T2D, CRP, BMI | 0.9299 | 0.5033 | 919 | 0.95 | 0.8 |
| rs8192675 | SCZ, T2D, CRP, BMI | 0.9299 | 0.5033 | 919 | 0.95 | 0.9 |
| rs8192675 | SCZ, T2D, CRP, BMI | 0.8434 | 0.5033 | 919 | 0.98 | 0.5 |
| rs8192675 | SCZ, T2D, CRP, BMI | 0.8434 | 0.5033 | 919 | 0.98 | 0.6 |
| rs8192675 | SCZ, T2D, CRP, BMI | 0.8434 | 0.5033 | 919 | 0.98 | 0.7 |
| rs8192675 | SCZ, T2D, CRP, BMI | 0.8434 | 0.5033 | 919 | 0.98 | 0.8 |
| rs8192675 | SCZ, CRP, BMI | 0.9093 | 0.351 | 919 | 0.98 | 0.9 |
| rs8192675 | SCZ, T2D, CRP, BMI | 0.7261 | 0.5033 | 919 | 0.99 | 0.5 |
| rs8192675 | SCZ, T2D, CRP, BMI | 0.7261 | 0.5033 | 919 | 0.99 | 0.6 |
| rs8192675 | SCZ, T2D, CRP, BMI | 0.7261 | 0.5033 | 919 | 0.99 | 0.7 |
| rs8192675 | SCZ, CRP, BMI | 0.8326 | 0.351 | 919 | 0.99 | 0.8 |
| rs8192675 | SCZ, BMI | 0.8957 | 0.6095 | 919 | 0.99 | 0.9 |
| rs8192675 | SCZ, BMI | 0.4097 | 0.6095 | 919 | 0.999 | 0.5 |
| rs17514846 | SCZ, CAD, FI | 0.9989 | 1.000 | 1071 | 0.95 | 0.5 |
| rs17514846 | SCZ, CAD, FI | 0.9989 | 1.000 | 1071 | 0.95 | 0.6 |
| rs17514846 | SCZ, CAD, FI | 0.9989 | 1.000 | 1071 | 0.95 | 0.7 |
| rs17514846 | SCZ, CAD, FI | 0.9989 | 1.000 | 1071 | 0.95 | 0.8 |
| rs17514846 | SCZ, CAD, FI | 0.9989 | 1.000 | 1071 | 0.95 | 0.9 |
| rs17514846 | SCZ, CAD, FI | 0.9971 | 1.000 | 1071 | 0.98 | 0.5 |
| rs17514846 | SCZ, CAD, FI | 0.9971 | 1.000 | 1071 | 0.98 | 0.6 |
| rs17514846 | SCZ, CAD, FI | 0.9971 | 1.000 | 1071 | 0.98 | 0.7 |
| rs17514846 | SCZ, CAD, FI | 0.9971 | 1.000 | 1071 | 0.98 | 0.8 |
| rs17514846 | SCZ, CAD, FI | 0.9971 | 1.000 | 1071 | 0.98 | 0.9 |
| rs17514846 | SCZ, CAD, FI | 0.9943 | 1.000 | 1071 | 0.99 | 0.5 |
| rs17514846 | SCZ, CAD, FI | 0.9943 | 1.000 | 1071 | 0.99 | 0.6 |
| rs17514846 | SCZ, CAD, FI | 0.9943 | 1.000 | 1071 | 0.99 | 0.7 |
| rs17514846 | SCZ, CAD, FI | 0.9943 | 1.000 | 1071 | 0.99 | 0.8 |
| rs17514846 | SCZ, CAD, FI | 0.9943 | 1.000 | 1071 | 0.99 | 0.9 |
| rs17514846 | SCZ, CAD, FI | 0.9457 | 1.000 | 1071 | 0.999 | 0.5 |
| rs17514846 | SCZ, CAD, FI | 0.9457 | 1.000 | 1071 | 0.999 | 0.6 |
| rs17514846 | SCZ, CAD, FI | 0.9457 | 1.000 | 1071 | 0.999 | 0.7 |
| rs17514846 | SCZ, CAD, FI | 0.9457 | 1.000 | 1071 | 0.999 | 0.8 |
| rs17514846 | SCZ, CAD, FI | 0.9457 | 1.000 | 1071 | 0.999 | 0.9 |
| rs13107325 | SCZ, TG, T2D, HDL, BMI | 0.8569 | 1.000 | 936 | 0.95 | 0.5 |
| rs13107325 | SCZ, TG, T2D, HDL, BMI | 0.8569 | 1.000 | 936 | 0.95 | 0.6 |
| rs13107325 | SCZ, TG, T2D, HDL, BMI | 0.8569 | 1.000 | 936 | 0.95 | 0.7 |
| rs13107325 | SCZ, TG, T2D, HDL, BMI | 0.8569 | 1.000 | 936 | 0.95 | 0.8 |
| rs13107325 | SCZ, TG, HDL, BMI | 0.9477 | 1.000 | 936 | 0.95 | 0.9 |
| rs13107325 | SCZ, TG, T2D, HDL, BMI | 0.7092 | 1.000 | 936 | 0.98 | 0.5 |
| rs13107325 | SCZ, TG, T2D, HDL, BMI | 0.7092 | 1.000 | 936 | 0.98 | 0.6 |
| rs13107325 | SCZ, TG, T2D, HDL, BMI | 0.7092 | 1.000 | 936 | 0.98 | 0.7 |
| rs13107325 | SCZ, TG, HDL, BMI | 0.8807 | 1.000 | 936 | 0.98 | 0.8 |
| rs13107325 | SCZ, TG, HDL, BMI | 0.8807 | 1.000 | 936 | 0.98 | 0.9 |
| rs13107325 | SCZ, TG, T2D, HDL, BMI | 0.5423 | 1.000 | 936 | 0.99 | 0.5 |
| rs13107325 | SCZ, TG, T2D, HDL, BMI | 0.5423 | 1.000 | 936 | 0.99 | 0.6 |
| rs13107325 | SCZ, TG, HDL, BMI | 0.7853 | 1.000 | 936 | 0.99 | 0.7 |
| rs13107325 | SCZ, TG, HDL, BMI | 0.7853 | 1.000 | 936 | 0.99 | 0.8 |
| rs13107325 | SCZ, HDL, BMI | 1.000 | 1.000 | 936 | 0.99 | 0.9 |
| rs13107325 | SCZ, HDL, BMI | 1.000 | 1.000 | 936 | 0.999 | 0.5 |
| rs13107325 | SCZ, HDL, BMI | 1.000 | 1.000 | 936 | 0.999 | 0.6 |
| rs13107325 | SCZ, HDL, BMI | 1.000 | 1.000 | 936 | 0.999 | 0.7 |
| rs13107325 | SCZ, HDL, BMI | 1.000 | 1.000 | 936 | 0.999 | 0.8 |
| rs13107325 | SCZ, HDL, BMI | 1.000 | 1.000 | 936 | 0.999 | 0.9 |
| rs3814883 | SCZ, BMI, T2D | 0.9885 | 0.9964 | 193 | 0.95 | 0.5 |
| rs3814883 | SCZ, BMI, T2D | 0.9885 | 0.9964 | 193 | 0.95 | 0.6 |
| rs3814883 | SCZ, BMI, T2D | 0.9885 | 0.9964 | 193 | 0.95 | 0.7 |
| rs3814883 | SCZ, BMI, T2D | 0.9885 | 0.9964 | 193 | 0.95 | 0.8 |
| rs3814883 | SCZ, BMI, T2D | 0.9885 | 0.9964 | 193 | 0.95 | 0.9 |
| rs3814883 | SCZ, BMI, T2D | 0.9717 | 0.9964 | 193 | 0.98 | 0.5 |
| rs3814883 | SCZ, BMI, T2D | 0.9717 | 0.9964 | 193 | 0.98 | 0.6 |
| rs3814883 | SCZ, BMI, T2D | 0.9717 | 0.9964 | 193 | 0.98 | 0.7 |
| rs3814883 | SCZ, BMI, T2D | 0.9717 | 0.9964 | 193 | 0.98 | 0.8 |
| rs3814883 | SCZ, BMI, T2D | 0.9717 | 0.9964 | 193 | 0.98 | 0.9 |
| rs3814883 | SCZ, BMI, T2D | 0.9449 | 0.9964 | 193 | 0.99 | 0.5 |
| rs3814883 | SCZ, BMI, T2D | 0.9449 | 0.9964 | 193 | 0.99 | 0.6 |
| rs3814883 | SCZ, BMI, T2D | 0.9449 | 0.9964 | 193 | 0.99 | 0.7 |
| rs3814883 | SCZ, BMI, T2D | 0.9449 | 0.9964 | 193 | 0.99 | 0.8 |
| rs3814883 | SCZ, BMI, T2D | 0.9449 | 0.9964 | 193 | 0.99 | 0.9 |
| rs3814883 | SCZ, BMI, T2D | 0.6316 | 0.9964 | 193 | 0.999 | 0.5 |
| rs3814883 | SCZ, BMI, T2D | 0.6316 | 0.9964 | 193 | 0.999 | 0.6 |
| rs12782894 | SCZ, BMI | 0.8834 | 0.6847 | 1255 | 0.95 | 0.5 |
| rs12782894 | SCZ, BMI | 0.8834 | 0.6847 | 1255 | 0.95 | 0.6 |
| rs12782894 | SCZ, BMI | 0.8834 | 0.6847 | 1255 | 0.95 | 0.7 |
| rs12782894 | SCZ, BMI | 0.8834 | 0.6847 | 1255 | 0.95 | 0.8 |
| rs12782894 | SCZ, BMI | 0.8834 | 0.6847 | 1255 | 0.95 | 0.9 |
| rs12782894 | SCZ, BMI | 0.7447 | 0.6847 | 1255 | 0.98 | 0.5 |
| rs12782894 | SCZ, BMI | 0.7447 | 0.6847 | 1255 | 0.98 | 0.6 |
| rs12782894 | SCZ, BMI | 0.7447 | 0.6847 | 1255 | 0.98 | 0.7 |
| rs12782894 | SCZ, BMI | 0.7447 | 0.6847 | 1255 | 0.98 | 0.8 |
| rs12782894 | SCZ, BMI | 0.5787 | 0.6847 | 1255 | 0.99 | 0.5 |
| rs12782894 | SCZ, BMI | 0.5787 | 0.6847 | 1255 | 0.99 | 0.6 |
| rs6265 | SCZ, TG, CAD, CRP, BMI, FI | 0.4796 | 0.8429 | 925 | 0.95 | 0.5 |
| rs6265 | SCZ, TG, CAD, CRP, BMI, FI | 0.4796 | 0.8429 | 925 | 0.95 | 0.6 |
| rs6265 | SCZ, CAD, CRP, BMI | 0.8607 | 0.7491 | 925 | 0.95 | 0.7 |
| rs6265 | SCZ, CAD, CRP, BMI | 0.8607 | 0.7491 | 925 | 0.95 | 0.8 |
| rs6265 | SCZ, CAD, BMI | 0.9552 | 0.8067 | 925 | 0.95 | 0.9 |
| rs6265 | SCZ, CAD, CRP, BMI | 0.7111 | 0.7491 | 925 | 0.98 | 0.5 |
| rs6265 | SCZ, CAD, CRP, BMI | 0.7111 | 0.7491 | 925 | 0.98 | 0.6 |
| rs6265 | SCZ, CAD, CRP, BMI | 0.7111 | 0.7491 | 925 | 0.98 | 0.7 |
| rs6265 | SCZ, CAD, BMI | 0.8951 | 0.8067 | 925 | 0.98 | 0.8 |
| rs6265 | SCZ, CAD, BMI | 0.8951 | 0.8067 | 925 | 0.98 | 0.9 |
| rs6265 | SCZ, CAD, CRP, BMI | 0.5406 | 0.7491 | 925 | 0.99 | 0.5 |
| rs6265 | SCZ, CAD, CRP, BMI | 0.5406 | 0.7491 | 925 | 0.99 | 0.6 |
| rs6265 | SCZ, CAD, BMI | 0.8071 | 0.8067 | 925 | 0.99 | 0.7 |
| rs6265 | SCZ, CAD, BMI | 0.8071 | 0.8067 | 925 | 0.99 | 0.8 |
| rs3800229 | SCZ, BMI, CAD | 0.8889 | 0.9519 | 872 | 0.95 | 0.5 |
| rs3800229 | SCZ, BMI, CAD | 0.8889 | 0.9519 | 872 | 0.95 | 0.6 |
| rs3800229 | SCZ, BMI, CAD | 0.8889 | 0.9519 | 872 | 0.95 | 0.7 |
| rs3800229 | SCZ, BMI, CAD | 0.8889 | 0.9519 | 872 | 0.95 | 0.8 |
| rs3800229 | SCZ, BMI, CAD | 0.8889 | 0.9519 | 872 | 0.95 | 0.9 |
| rs3800229 | SCZ, BMI, CAD | 0.7546 | 0.9519 | 872 | 0.98 | 0.5 |
| rs3800229 | SCZ, BMI, CAD | 0.7546 | 0.9519 | 872 | 0.98 | 0.6 |
| rs3800229 | SCZ, BMI, CAD | 0.7546 | 0.9519 | 872 | 0.98 | 0.7 |
| rs3800229 | SCZ, BMI, CAD | 0.7546 | 0.9519 | 872 | 0.98 | 0.8 |
| rs3800229 | SCZ, BMI, CAD | 0.5909 | 0.9519 | 872 | 0.99 | 0.5 |
| rs3800229 | SCZ, BMI, CAD | 0.5909 | 0.9519 | 872 | 0.99 | 0.6 |
| rs3800229 | SCZ, BMI, CAD | 0.5909 | 0.9519 | 872 | 0.99 | 0.7 |
| rs2239647 | SCZ, T2D, BMI, CAD | 0.7872 | 0.6625 | 1584 | 0.95 | 0.6 |
| rs2239647 | SCZ, T2D, BMI, CAD | 0.7872 | 0.6625 | 1584 | 0.95 | 0.7 |
| rs2239647 | SCZ, T2D, BMI, CAD | 0.7872 | 0.6625 | 1584 | 0.95 | 0.8 |
| rs2239647 | SCZ, BMI | 0.9883 | 0.6317 | 1584 | 0.95 | 0.9 |
| rs2239647 | SCZ, T2D, BMI, CAD | 0.5916 | 0.6625 | 1584 | 0.98 | 0.5 |
| rs2239647 | SCZ, T2D, BMI, CAD | 0.5916 | 0.6625 | 1584 | 0.98 | 0.6 |
| rs2239647 | SCZ, BMI | 0.9712 | 0.6317 | 1584 | 0.98 | 0.7 |
| rs2239647 | SCZ, BMI | 0.9712 | 0.6317 | 1584 | 0.98 | 0.8 |
| rs2239647 | SCZ, BMI | 0.9712 | 0.6317 | 1584 | 0.98 | 0.9 |
| rs2239647 | SCZ, BMI | 0.944 | 0.6317 | 1584 | 0.99 | 0.5 |
| rs2239647 | SCZ, BMI | 0.944 | 0.6317 | 1584 | 0.99 | 0.6 |
| rs2239647 | SCZ, BMI | 0.944 | 0.6317 | 1584 | 0.99 | 0.7 |
| rs2239647 | SCZ, BMI | 0.944 | 0.6317 | 1584 | 0.99 | 0.8 |
| rs2239647 | SCZ, BMI | 0.944 | 0.6317 | 1584 | 0.99 | 0.9 |
| rs2239647 | SCZ, BMI | 0.6234 | 0.6317 | 1584 | 0.999 | 0.5 |
| rs2239647 | SCZ, BMI | 0.6234 | 0.6317 | 1584 | 0.999 | 0.6 |
| rs11191514 | SCZ, CAD, BMI | 0.7651 | 0.297 | 710 | 0.95 | 0.5 |
| rs11191514 | SCZ, CAD, BMI | 0.7651 | 0.297 | 710 | 0.95 | 0.6 |
| rs11191514 | SCZ, CAD, BMI | 0.7651 | 0.297 | 710 | 0.95 | 0.7 |
| rs11191514 | SCZ, CAD, BMI | 0.5695 | 0.297 | 710 | 0.98 | 0.5 |
| rs6031855 | SCZ, BMI | 0.5877 | 0.2771 | 990 | 0.95 | 0.5 |
| rs6031855 | SCZ, BMI | 0.5877 | 0.2771 | 990 | 0.95 | 0.6 |
| rs6031855 | SCZ, BMI | 0.5877 | 0.2771 | 990 | 0.95 | 0.7 |
| rs6031855 | SCZ, BMI | 0.3224 | 0.2771 | 990 | 0.98 | 0.5 |

SCZ=schizophrenia; BMI=body mass index; CAD=coronary artery disease; HDL=high-density lipoprotein; TG=triglycerides; LDL=low-density lipoprotein; T2D=type 2 diabetes; FI=fasting insulin; CRP=C-reactive protein.^1^PP_coloc_ indicates posterior probability of single shared causal SNP at default prior and threshold settings. ^2^PP_explained_ corresponds to amount of shared variance explained by the candidate SNP. ^3^Corresponds to the number of SNPs present in all datasets

**Supplementary Figures**

**Supplementary Figure 1: Whole Genome Correlations between Schizophrenia, Cardiometabolic and Inflammatory Traits**

**
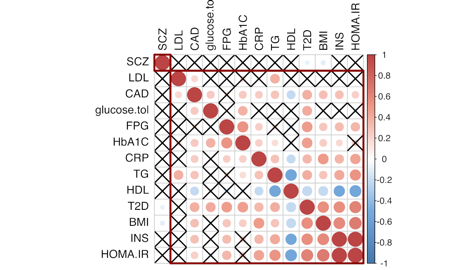
**

X indicates correlations that did not meet Bonferroni-corrected evidential threshold (*p*=0.004). Hierarchical clusters indicated with red boxes. SCZ=schizophrenia; LDL=low-density lipoprotein; CAD=coronary artery disease; glucose.tol=two hour glucose; FPG=fasting plasma glucose; HbA1C=glycated haemoglobin; CRP=C-reactive protein; TG=triglycerides; HDL=high-density lipoprotein; T2D=type 2 diabetes mellitus; BMI=body mass index; INS=fasting insulin; HOMA.IR=homeostatic model assessment for insulin resistance.

**Supplementary Figure 2: Regions of Local Genetic Correlation between Schizophrenia and Cardiometabolic and Inflammatory Traits**

1. Fasting Insulin

1. Type 2 Diabetes
2. HDL
3. Triglycerides
4. LDL
5. CRP
6. Coronary Artery Disease

**Supplementary** **Figure 3: Regional Genetic Association Plots for Seven Loci Indicating Evidence for Colocalization between Schizophrenia, Cardiometabolic and Inflammatory Traits**

1.
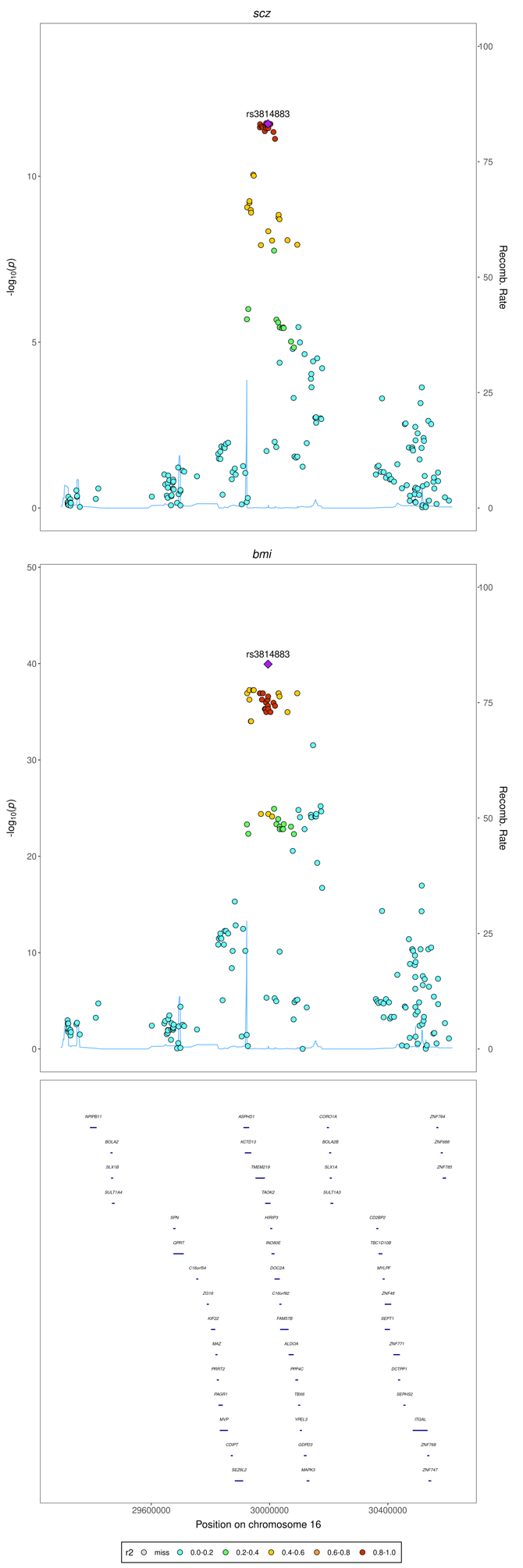
**rs3814883 – *TAOK2***
2.
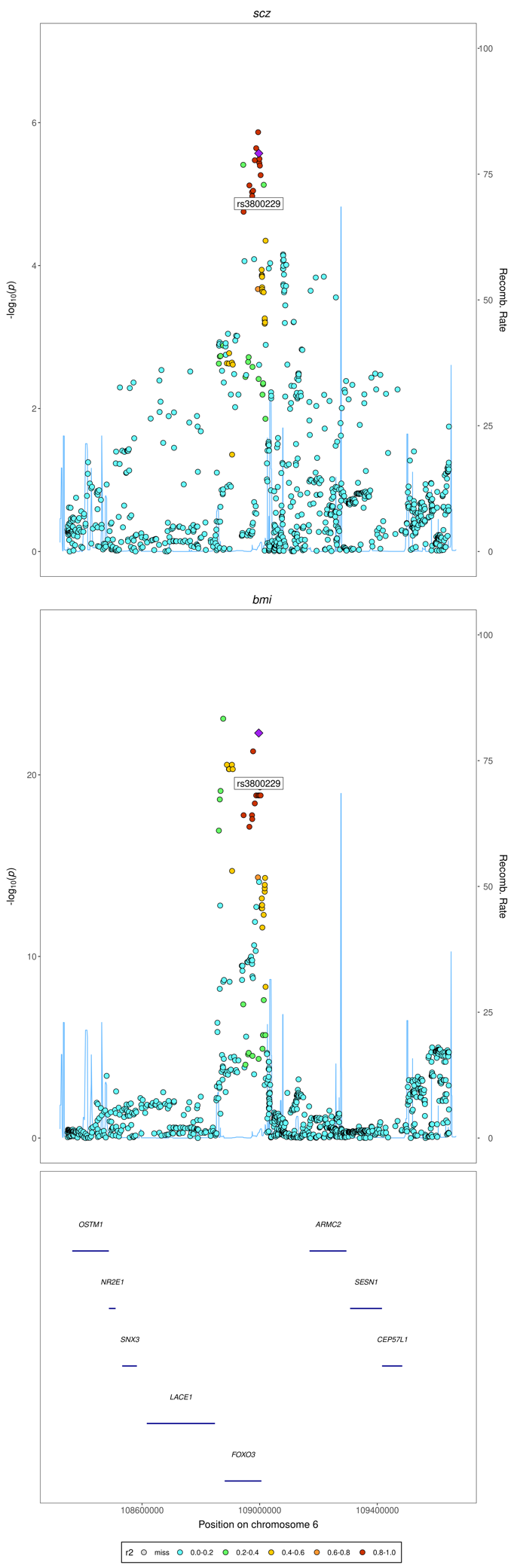
**rs3800229 – *FOXO3***
3. **
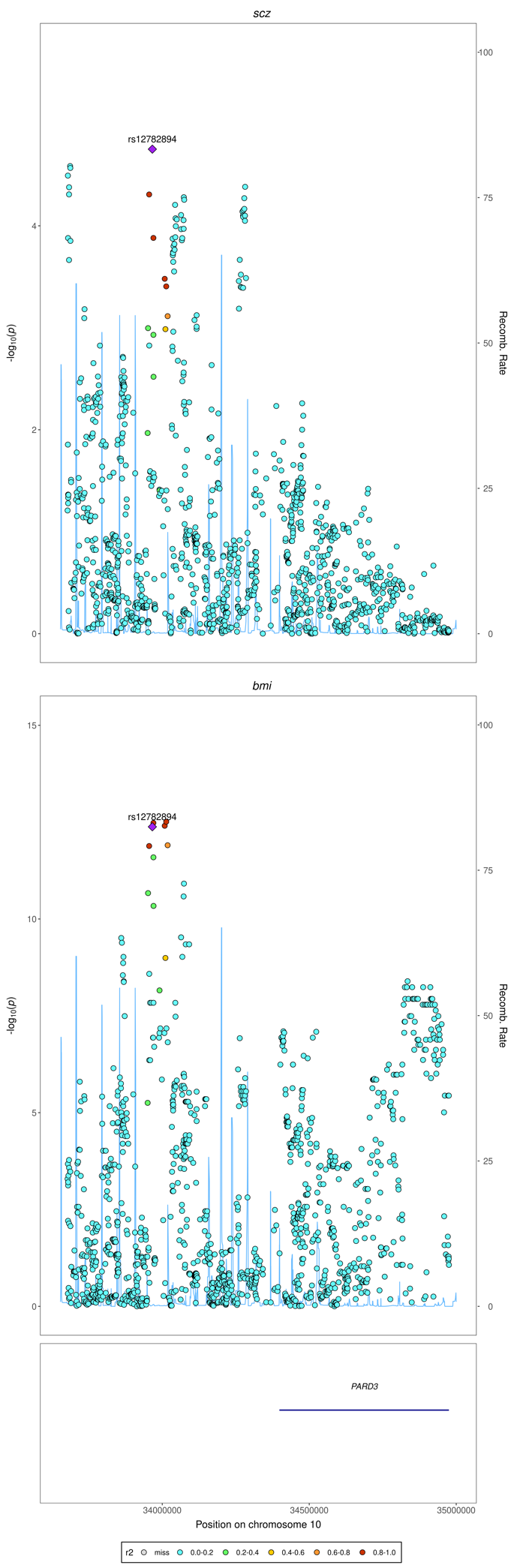
rs12782894**
4. **
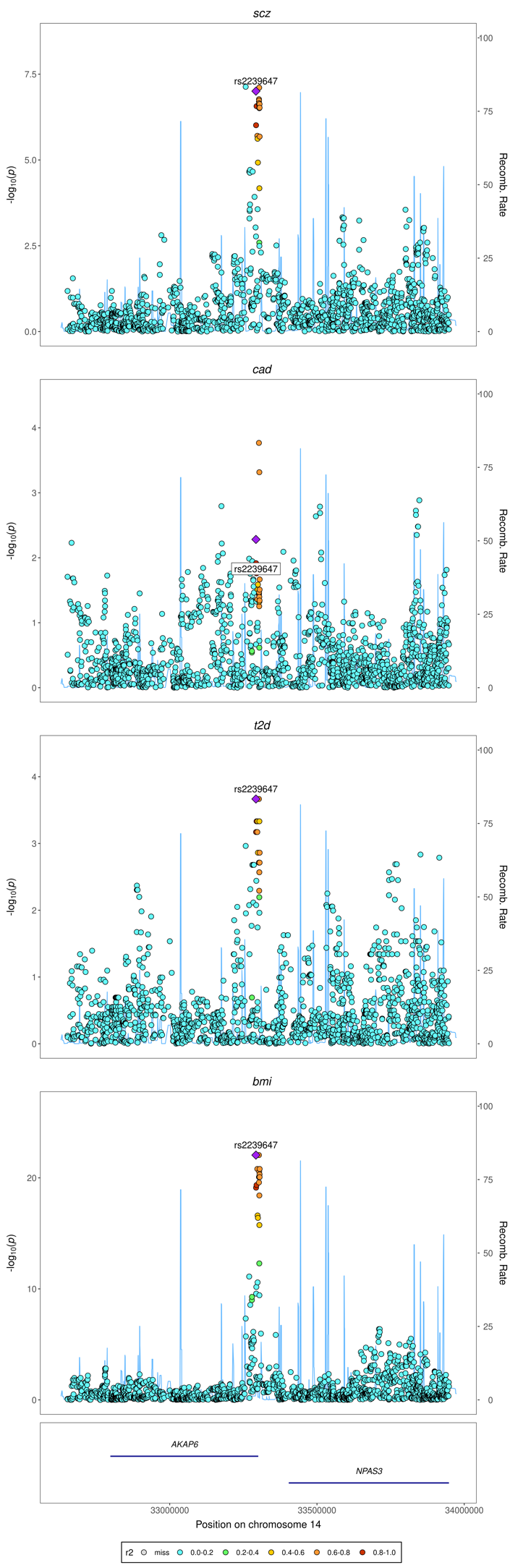
rs2239647 – *AKAP6***
5. **
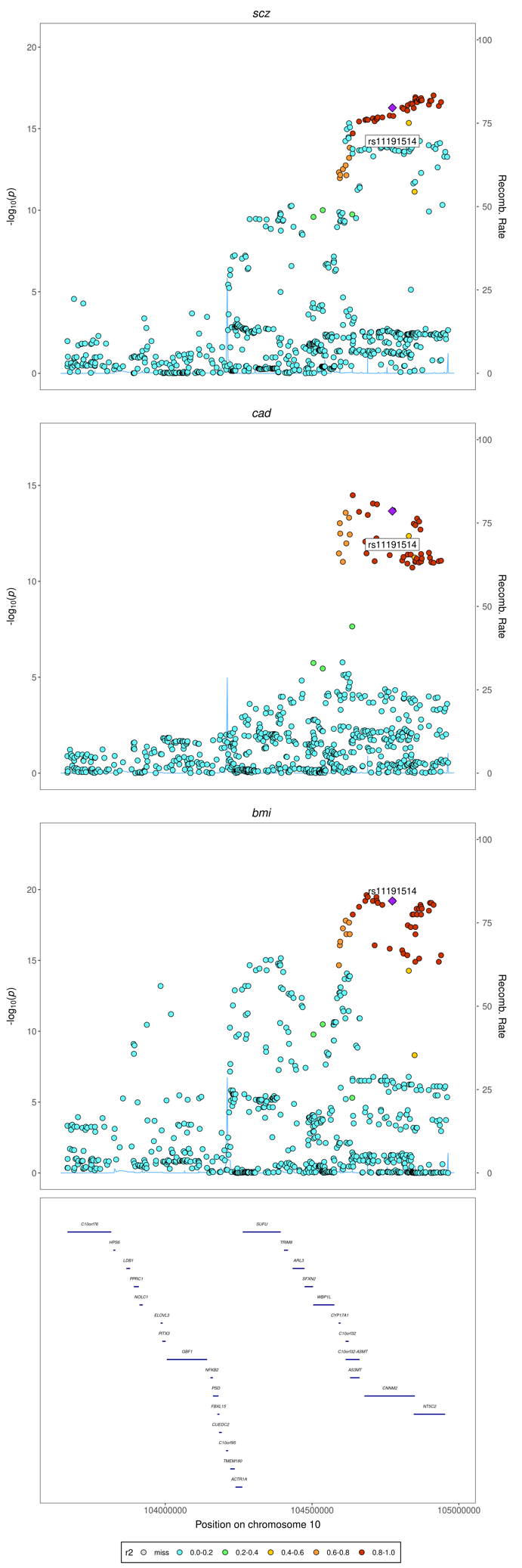
rs1191514 – *CNNM2***
6. **
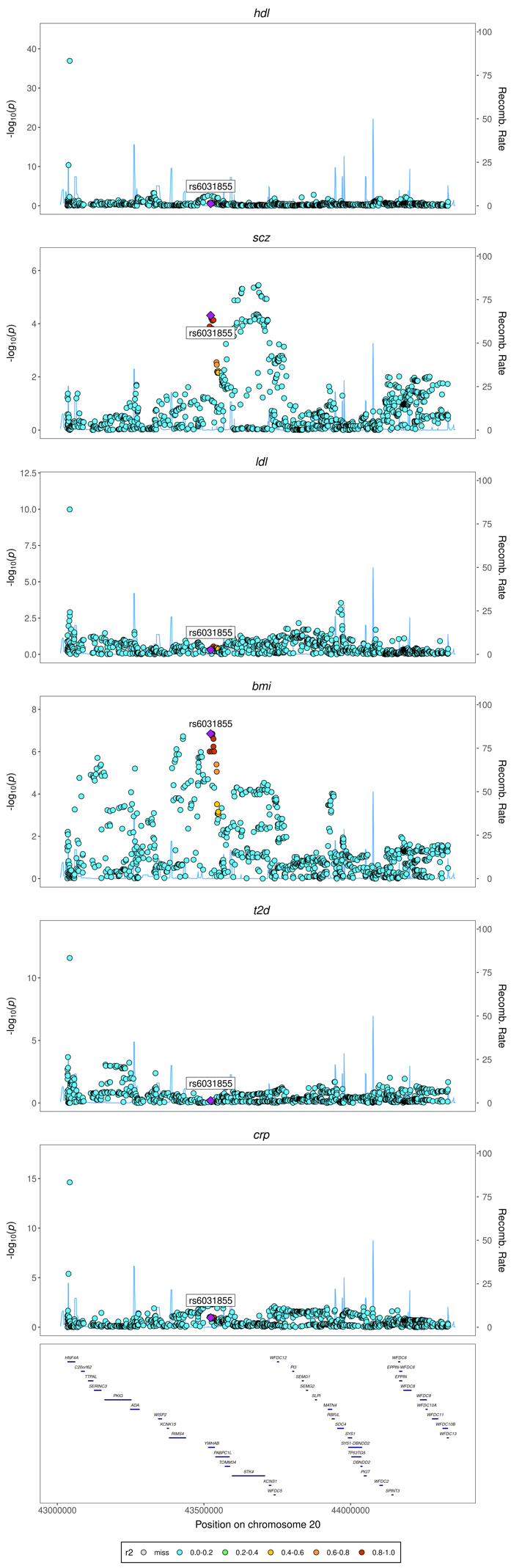
rs6031855 - *YWHAB***

Regional association plots denoting chromosomal location (x axis) and strength of association with listed trait (-log_10(p)_) (y axis). SNP r^2^ estimated from the EPIC-Norfolk cohort.

**Supplementary Figure 4: Heatmap Sensitivity Plots for SNPs with Evidence of Colocalization Between Schizophrenia and Cardiometabolic and Inflammatory Traits**

1. **rs8192675 – *SLC2A2***

1. **rs17514846 - *FURIN***

1. **rs13107325 – *SLC39A8***

1. **rs3814883 – *TAOK2***

1. **rs12782894**

1. **rs6265 - *BDNF***

1. **rs3800229 – *FOXO3***

1. **rs2239647 – *AKAP6***

1. **rs11191514 – *CNNM2***

1. **rs6031855 - *YWHAB***

Heatmaps drawn based on a similarity matrix across increasingly stringent prior and threshold permutations, from prior1 = 1x10^-4^; prior2 = 0.05; regional/alignment thresholds = 0.5, to prior2 = 0.001; regional/alignment thresholds = 0.9).
1 = evidence of colocalization across all permutations (dark red) and 0 = no evidence of colocalization at any permutation (beige). bmi=body mass index; hdl = high-density lipoprotein; tg=triglycerides; scz=schizophrenia; ldl=low-density lipoprotein; t2d=type 2 diabetes mellitus; crp=C-reactive protein; fi=fasting insulin; cad=coronary artery disease.
